# A fast combination method in DSmT and its application to recommender system

**DOI:** 10.1371/journal.pone.0189703

**Published:** 2018-01-19

**Authors:** Yilin Dong, Xinde Li, Yihai Liu

**Affiliations:** 1 Key Laboratory of Measurement and Control of CSE, Ministry of Education, School of Automation, Southeast University, Nanjing, Jiangsu Province, China; 2 Jiangsu Automation Research Institute, Lianyungang, Jiangsu Province, China; Southwest University, CHINA

## Abstract

In many applications involving epistemic uncertainties usually modeled by belief functions, it is often necessary to approximate general (non-Bayesian) basic belief assignments (BBAs) to subjective probabilities (called Bayesian BBAs). This necessity occurs if one needs to embed the fusion result in a system based on the probabilistic framework and Bayesian inference (e.g. tracking systems), or if one needs to make a decision in the decision making problems. In this paper, we present a new fast combination method, called modified rigid coarsening (MRC), to obtain the final Bayesian BBAs based on hierarchical decomposition (coarsening) of the frame of discernment. Regarding this method, focal elements with probabilities are coarsened efficiently to reduce computational complexity in the process of combination by using disagreement vector and a simple dichotomous approach. In order to prove the practicality of our approach, this new approach is applied to combine users’ soft preferences in recommender systems (RSs). Additionally, in order to make a comprehensive performance comparison, the proportional conflict redistribution rule #6 (PCR6) is regarded as a baseline in a range of experiments. According to the results of experiments, MRC is more effective in accuracy of recommendations compared to original Rigid Coarsening (RC) method and comparable in computational time.

## Introduction

The theory of belief functions, known as Dempster-Shafer Theory (DST) was developed by Shafer [1] in 1976 from Dempster’s works [2]. Belief functions allow one to model epistemic uncertainty [3] and they have been already used in many applications since the 1990’s [4], mainly those relevant to expert systems, decision-making support and information fusion. To palliate some limitations (such as high computational compelxity) of DST, Dezert and Smarandache proposed an extended mathematical framework of belief functions with new efficient quantitative and qualitative rules of combinations, which was called DSmT (Dezert and Smarandache Theory) in literature [5, 6] with applications listed in [7]. One of the major drawbacks of DST and DSmT is their high computational complexities, on condition that the fusion space (i.e. frame of discernment—FoD) and the number of sources to combine are large. DSmT is more complex than DST, and the Proportional Conflict Redistribution rule #6 (PCR6 rule) becomes computationally intractable in the worst case as soon as the cardinality of the Frame of Discernment (FoD) is greater than six.

To reduce the computational cost of operations with belief functions when the number of focal elements is very large, several approaches have been proposed by different authors. Basically, the existing approaches rely either on efficient implementations of computations as proposed for instance in [8, 9], or on approximation techniques of original Basic Belief Assignment (BBA) to combine [10–14], or both. From a fusion standpoint, two approaches are usually adopted: 1) one can approximate at first each BBA in subjective probabilities and use Bayes fusion rule to get the final Bayesian BBA [11, 12], or 2) one can fuse all the BBAs with a fusion rule, typically Dempster-Shafer’s, or proportional conflict redistribution rule #6 (PCR6) rules (which is very costly in computations), and convert the combined BBA in a subjective probability measure [10, 14]. The former method is the simplest method but it generates a high loss of information included in the original BBAs, whereas the latter method is intractable for high dimension issues.

This paper presents a new combination method, called modified rigid coarsening (MRC), to get the final Bayesian BBAs based on hierarchical decomposition (coarsening) of the frame of discernment, which can be seen as an intermediary approach between the two aforementioned methods. This hierarchical structure allows to encompass bintree decomposition and mass of coarsening FoD on it. To prove the practicality of our proposed method, MRC is applied to combine users’ preferences so as to provide the suitable recommendation for RSs. Preliminary work on original rigid coarsening (RC) has been published in our recent work [15] (This is an extended version of the paper presented at the 20th IEEE International Conference on Information Fusion, XIAN, China). In this paper, more detailed analyses of this new combination method are provided. More importantly, this innovative method is also applied into the real application. These are all added values (contributions) of this paper.

The main contributions of this paper are:

1. the presentation of the FoD bintree decomposition on which will be done the BBAs approximations;
2. user preferences in Recommender Systems (RSs) are modeled by DSmT-Modeling Function.

In order to measure the efficiency and effectiveness of the MRC, it is integrated in the RSs based on DSmT and compared to traditional methods in the experiments. The results show that regarding the accuracy of recommendations, MRC is extremely close to classical PCR6; and the computational time of MRC can be obviously superior to that of PCR6.

The remainder of this paper is organized as follows. In section 2, we review relevant prior work on DST and DSmT first. In section 3, MRC is presented. In section 4, a recommendation system based on DSmT, that employs MRC to combine users’ preferences, is shown. In section 5, we evaluate our proposed algorithm based on two public datasets: Movielens and Flixster. Finally, we conclude and discuss future work.

## Mathematical background

This section provides a brief reminder of the basics of DST and DSmT, which is necessary for the presentation and understanding of the more general MRC of Section 3.

In DST framework, the frame of discernment (Here, we use the symbol ≜ to mean equals by definition.) $\Theta ≜\{\theta_{1},\ldots ,\theta_{n}\}$ (*n* ≥ 2) is a set of exhaustive and exclusive elements (hypotheses) which represents the possible solutions of the problem under consideration and thus Shafer’s model assumes *θ*_*i*_ ∩ *θ*_*j*_ = ∅ for *i* ≠ *j* in {1, …, *n*}. A basic belief assignment (BBA) *m*(⋅) is defined by the mapping: 2^Θ^ ↦ [0, 1], verifying *m*(∅) = 0 and ∑_*A*∈2^Θ^_ *m*(*A*) = 1. In DSmT, one can abandon Shafer’s model (if Shafer’s model doesn’t fit with the problem) and refute the principle of the third excluded middle. The third excluded middle principle assumes the existence of the complement for any elements/propositions belonging to the power set 2^Θ^. Instead of defining the BBAs on the power set $2^{\Theta }≜\left(\Theta ,\cup \right)$ of the FoD, the BBAs are defined on the so-called *hyper-power set* (or Dedekind’s lattice) denoted $D^{\Theta }≜\left(\Theta ,\cup ,\cap \right)$ whose cardinalities follows Dedekind’s numbers sequence, see [6], Vol.1 for details and examples. A (generalized) BBA, called a mass function, *m*(⋅) is defined by the mapping: *D*^Θ^ ↦ [0, 1], verifying *m*(∅) = 0 and ∑_*A*∈*D*^Θ^_ *m*(*A*) = 1. The DSmT framework encompasses DST framework because 2^Θ^ ⊂ *D*^Θ^. In DSmT, we can take into account also a set of *integrity constraints* on the FoD (if known), by specifying all the pairs of elements which are really disjoint. Stated otherwise, Shafer’s model is a specific DSm model where all elements are deemed to be disjoint. *A* ∈ *D*^Θ^ is called a focal element of *m*(.) if *m*(*A*) > 0. A BBA is called a Bayesian BBA if all of its focal elements are singletons and Shafer’s model is assumed, otherwise it is called non-Bayesian [1]. A full ignorance source is represented by the vacuous BBA *m*_*v*_(Θ) = 1. The belief (or credibility) and plausibility functions are respectively defined by $Bel\left(X\right)≜\sum_{Y\in D^{\Theta }|Y\subseteq X}m\left(Y\right)$ and $Pl\left(X\right)≜\sum_{Y\in D^{\Theta }|Y\cap X\neq \emptyset }m\left(Y\right)$. BI(X)≜[Bel(X),Pl(X)] is called the belief interval of *X*. Its length U(X)≜Pl(X)-Bel(X) measures the degree of uncertainty of *X*.

In 1976, Shafer did propose Dempster’s rule and we use DS index to refer to Dempster-Shafer’s rule (DS rule) because Shafer did really promote Dempster’s rule in in his milestone book [1]) to combine BBAs in DST framework. DS rule is defined by *m*_*DS*_(∅) = 0 and ∀*A* ∈ 2^Θ^\{∅},

$$\begin{array}{c} m_{DS}\left(A\right)=\frac{\sum_{B,C\in 2^{\Theta }|B\cap C=A}m_{1}\left(B\right)m_{2}\left(C\right)}{1-\sum_{B,C\in 2^{\Theta }|B\cap C=\emptyset }m_{1}\left(B\right)m_{2}\left(C\right)} \end{array}$$ (1)

The DS rule formula is commutative and associative and can be easily extended to the fusion of *S* > 2 BBAs. Unfortunately, DS rule has been highly disputed during the last decades by many authors because of its counter-intuitive behavior in high or even low conflict situations, and that is why many rules of combination were proposed in literature to combine BBAs [16]. To palliate DS rule drawbacks, the very interesting PCR6 was proposed in DSmT and it is usually adopted (PCR6 rule coincides with PCR5 when combining only two BBAs [6]) in recent applications of DSmT. The fusion of two BBAs *m*_1_(.) and *m*_2_(.) by the PCR6 rule is obtained by *m*_*PCR*6_(∅) = 0 and ∀*A* ∈ *D*^Θ^\{∅}

$$\begin{array}{ccc} m_{PCR6}\left(A\right) & = & m_{12}\left(A\right)+\\ & & \sum_{B\in D^{\Theta }\setminus \left\{A\right\}|A\cap B=\emptyset }\left[\frac{m_{1}{\left(A\right)}^{2}m_{2}\left(B\right)}{m_{1}\left(A\right)+m_{2}\left(B\right)}+\frac{m_{2}{\left(A\right)}^{2}m_{1}\left(B\right)}{m_{2}\left(A\right)+m_{1}\left(B\right)}\right] \end{array}$$ (2)

where *m*_12_(*A*) = ∑_*B*,*C*∈*D*^Θ^|*B*∩*C*=*A*_*m*_1_(*B*)*m*_2_(*C*) is the conjunctive operator, and each element *A* and *B* are expressed in their disjunctive normal form. If the denominator involved in the fraction is zero, then this fraction is discarded. The general PCR6 formula for combining more than two BBAs altogether is given in [6], Vol. 3. We adopt the generic notation $m_{12}^{PCR6}\left(.\right)=PCR6\left(m_{1}\left(.\right),m_{2}\left(.\right)\right)$ to denote the fusion of *m*_1_(.) and *m*_2_(.) by PCR6 rule. PCR6 is not associative and PCR6 rule can also be applied in DST framework (with Shafer’s model of FoD) by replacing *D*^Θ^ by 2^Θ^ in Eq (2).

## Modified rigid coarsening for fusion of Bayesian BBAs

Here, we introduce the principle of MRC of FoD to reduce the computational complexity of PCR6 combination of original Bayesian BBAs. Considering the case of non-Bayesian BBAs, it requires decoupling all non-singletons in these BBAs in advance, The fusion of original non-Bayesian BBAs needs to be decoupled by using DSmP in advance, which will explain in Section 4.

### Rigid coarsening

This proposal was initially called *rigid coarsening (RC)* in our previous works [17–19] and currently improved in our recent work [15]. The goal of this coarsening is to replace the original (refined) FoD Θ by a set of coarsened ones to make computation of the PCR6 rule tractable. Because we consider here only Bayesian BBA to combine, their focal elements are only singletons of the FoD $\Theta ≜\{\theta_{1},\ldots ,\theta_{n}\}$, with *n* ≥ 2, and we assume Shafer’s model of the FoD Θ. A coarsening of the FoD Θ means to replace it with another FoD less specific of smaller dimension Ω = {*ω*_1_, …, *ω*_*k*_} with *k* < *n* from the elements of Θ. This can be done in many ways depending the problem under consideration. Generally, the elements of Ω are singletons of Θ, and disjunctions of elements of Θ. For example, if Θ = {*θ*_1_, *θ*_2_, *θ*_3_, *θ*_4_}, then a possible coarsened frame built from Θ could be, for instance, Ω = {*ω*_1_ = *θ*_1_, *ω*_2_ = *θ*_2_, *ω*_3_ = *θ*_3_ ∪ *θ*_4_}, or Ω = {*w*_1_ = *θ*_1_ ∪ *θ*_2_, *ω*_2_ = *θ*_3_ ∪ *θ*_4_}, etc.

**Definition 1**: *When dealing with Bayesian BBAs, the projection (For clarity and convenience, we put explicitly as upper index the FoD for which the belief mass refers)* *m*^Ω^(.) *of the original BBA* *m*^Θ^(.) *is simply obtained by taking*

$$\begin{array}{c} m^{\Omega }\left(\omega_{i}\right)=\sum_{\theta_{j}\subseteq \omega_{i}}m^{\Theta }\left(\theta_{j}\right) \end{array}$$ (3)

The rigid coarsening process is a simple dichotomous approach of coarsening obtained as follows:

- If *n* = |Θ| is an even number:
  The disjunction of the *n*/2 first elements *θ*_1_ to $\theta_{\frac{n}{2}}$ of Θ define the element *ω*_1_ of Ω, and the last *n*/2 elements $\theta_{\frac{n}{2}+1}$ to *θ*_*n*_ of Θ define the element *ω*_2_ of Ω, that is
  $$\begin{array}{c} \Omega ≜\{\omega_{1}=\theta_{1}\cup \ldots \cup \theta_{\frac{n}{2}},\omega_{2}=\theta_{\frac{n}{2}+1}\cup \ldots \cup \theta_{n}\} \end{array}$$
  and based on Eq (3), one has
  $$\begin{array}{c} m^{\Omega }\left(\omega_{1}\right)=\sum_{j=1,\ldots ,\frac{n}{2}}m^{\Theta }\left(\theta_{j}\right) \end{array}$$ (4)
  $$\begin{array}{c} m^{\Omega }\left(\omega_{2}\right)=\sum_{j=\frac{n}{2}+1,\ldots ,n}m^{\Theta }\left(\theta_{j}\right) \end{array}$$ (5)
  For example, if Θ = {*θ*_1_, *θ*_2_, *θ*_3_, *θ*_4_}, and one considers the Bayesian BBA *m*^Θ^(*θ*_1_) = 0.1, *m*^Θ^(*θ*_2_) = 0.2, *m*^Θ^(*θ*_3_) = 0.3 and *m*^Θ^(*θ*_4_) = 0.4, then Ω = {*ω*_1_ = *θ*_1_ ∪ *θ*_2_, *ω*_2_ = *θ*_3_ ∪ *θ*_4_} and *m*^Ω^(*ω*_1_) = 0.1 + 0.2 = 0.3 and *m*^Ω^(*ω*_2_) = 0.3 + 0.4 = 0.7.
- If *n* = |Θ| is an odd number:
  In this case, the element *ω*_1_ of the coarsened frame Ω is the disjunction of the [*n*/2 + 1] (The notation [*x*] means the integer part of *x*) first elements of Θ, and the element *ω*_2_ is the disjunction of other elements of Θ. That is
  $$\begin{array}{c} \Omega ≜\{\omega_{1}=\theta_{1}\cup \ldots \cup \theta_{\left[\frac{n}{2}+1\right]},\omega_{2}=\theta_{[\frac{n}{2}+1]+1}\cup \ldots \cup \theta_{n}\} \end{array}$$
  and based on Eq (3), one has
  $$\begin{array}{c} m^{\Omega }\left(\omega_{1}\right)=\sum_{j=1,\ldots ,[\frac{n}{2}+1]}m^{\Theta }\left(\theta_{j}\right) \end{array}$$ (6)
  $$\begin{array}{c} m^{\Omega }\left(\omega_{2}\right)=\sum_{j=[\frac{n}{2}+1]+1,\ldots ,n}m^{\Theta }\left(\theta_{j}\right) \end{array}$$ (7)
  For example, if Θ = {*θ*_1_, *θ*_2_, *θ*_3_, *θ*_4_, *θ*_5_}, and one considers the Bayesian BBA *m*^Θ^(*θ*_1_) = 0.1, *m*^Θ^(*θ*_2_) = 0.2, *m*^Θ^(*θ*_3_) = 0.3, *m*^Θ^(*θ*_4_) = 0.3 and *m*^Θ^(*θ*_5_) = 0.1, then Ω = {*ω*_1_ = *θ*_1_ ∪ *θ*_2_ ∪ *θ*_3_, *ω*_2_ = *θ*_4_ ∪ *θ*_5_} and *m*^Ω^(*ω*_1_) = 0.1 + 0.2 + 0.3 = 0.6 and *m*^Ω^(*ω*_2_) = 0.3 + 0.1 = 0.4.

Of course, the same coarsening strategy applies to all original BBAs $m_{s}^{\Theta }\left(.\right)$, *s* = 1, …*S* of the *S* > 1 sources of evidence to work with less specific BBAs $m_{s}^{\Omega }\left(.\right)$, *s* = 1, …*S*. The less specific BBAs (called coarsened BBAs by abuse of language) can then be combined with the PCR6 rule of combination according to formula Eq (2). This dichotomous coarsening method is repeated iteratively *l* times as schematically represented by a bintree. Here, we consider bintree only for simplicity, which means that the coarsened frame Ω consists of two elements only. Of course a similar method can be used with tri-tree, quad-tree, etc. The last step of this hierarchical process is to calculate the combined (Bayesian) BBA of all focal elements according to the connection weights of the bintree structure, where the number of layers *l* of the tree depends on the cardinality |Θ| of the original FoD Θ. Specifically, the mass of each focal element is updated depending on the connection weights of link paths from root to terminal nodes. This principle is illustrated in details in the following example.

**Example 1:** Let’s consider Θ = {*θ*_1_, *θ*_2_, *θ*_3_, *θ*_4_, *θ*_5_}, and the following three Bayesian BBAs can be seen in Table 1:

**Table 1:** Three Bayesian BBAs for Example 1.

Focal elem.	$m_{1}^{\Theta }\left(.\right)$	$m_{2}^{\Theta }\left(.\right)$	$m_{3}^{\Theta }\left(.\right)$
*θ*_1_	0.1	0.4	0
*θ*_2_	0.2	0	0.1
*θ*_3_	0.3	0.1	0.5
*θ*_4_	0.3	0.1	0.4
*θ*_5_	0.1	0.4	0

**Fig 1:**
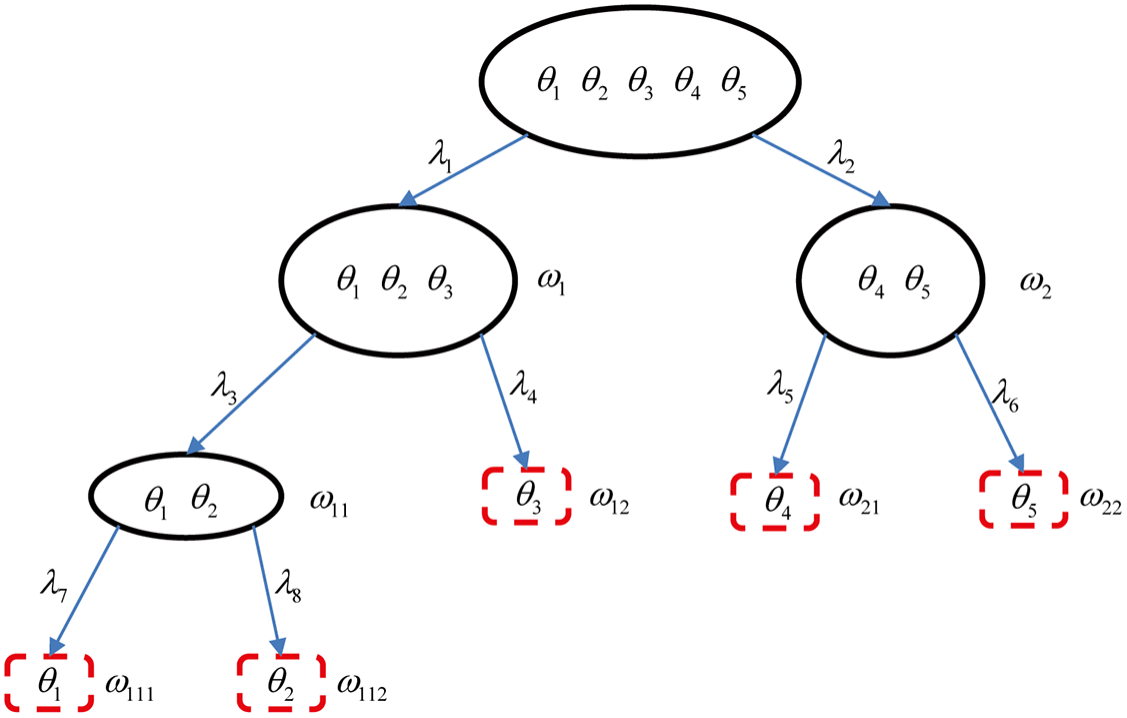
Fusion of Bayesian BBAs using bintree coarsening for Example 1.

The rigid coarsening and fusion of BBAs is deduced from the following steps:

**Step 1**: We define the bintree structure based on iterative half split of FoD as shown in Fig 1.

The connecting weights are denoted as λ_1_, …, λ_8_. The elements of the frames Ω_*l*_ are defined as follows:

- At layer *l* = 1: $\Omega_{1}=\left\{\omega_{1}≜\theta_{1}\cup \theta_{2}\cup \theta_{3},\omega_{2}≜\theta_{4}\cup \theta_{5}\right\}$
- At layer *l* = 2:
  $$\begin{array}{c} \Omega_{2}=\left\{\omega_{11}≜\theta_{1}\cup \theta_{2},\omega_{12}≜\theta_{3},\omega_{21}≜\theta_{4},\omega_{22}=\theta_{5}\right\} \end{array}$$
- At layer *l* = 3: $\Omega_{3}=\left\{\omega_{111}≜\theta_{1},\omega_{112}≜\theta_{2}\right\}$

**Step 2**: The BBAs of elements of the (sub-) frames Ω_*l*_ are obtained as follows:

- At layer *l* = 1, we use Eqs (6) and (7) because |Θ| = 5 is an odd number. Therefore, we get the BBAs in Table 2:
- At layer *l* = 2: We work with the two subframes $\Omega_{21}≜\left\{\omega_{11},\omega_{12}\right\}$ and $\Omega_{22}≜\left\{\omega_{21},\omega_{22}\right\}$ of Ω_2_ with the BBAs in Tables 3 and 4:
  These mass values are obtained by the proportional redistribution of the mass of each focal element with respect to the mass of its parent focal element in the bin tree. For example, $m_{2}^{\Omega_{21}}\left(\omega_{11}\right)=4/5$ is derived by taking
  $$\begin{array}{c} m_{2}^{\Omega_{21}}\left(\omega_{11}\right)=\frac{m_{2}^{\Theta }\left(\theta_{1}\right)+m_{2}^{\Theta }\left(\theta_{2}\right)}{m_{2}^{\Theta }\left(\theta_{1}\right)+m_{2}^{\Theta }\left(\theta_{2}\right)+m_{2}^{\Theta }\left(\theta_{3}\right)}=\frac{0.4}{0.5}=\frac{4}{5} \end{array}$$
  Other masses of coarsening focal elements are computed similarly using this proportional redistribution method.
- At layer *l* = 3: We use again the proportional redistribution method which gives us the BBAs of the sub-frames Ω_3_ in Table 5:

**Table 2:** The BBAs of elements of the sub-frames Ω_1_ for Example 1.

Focal elem.	$m_{1}^{\Theta }\left(.\right)$	$m_{2}^{\Theta }\left(.\right)$	$m_{3}^{\Theta }\left(.\right)$
$\omega_{1}≜\theta_{1}\cup \theta_{2}\cup \theta_{3}$	0.6	0.5	0.6
$\omega_{2}≜\theta_{4}\cup \theta_{5}$	0.4	0.5	0.4

**Table 3:** The BBAs of elements of the sub-frames Ω_21_ for Example 1.

Focal elem.	$m_{1}^{\Omega_{21}}\left(.\right)$	$m_{2}^{\Omega_{21}}\left(.\right)$	$m_{3}^{\Omega_{21}}\left(.\right)$
$\omega_{11}≜\theta_{1}\cup \theta_{2}$	$\frac{1}{2}$	$\frac{4}{5}$	$\frac{1}{6}$
$\omega_{12}≜\theta_{3}$	$\frac{1}{2}$	$\frac{1}{5}$	$\frac{5}{6}$

**Table 4:** The BBAs of elements of the sub-frames Ω_22_ for Example 1.

Focal elem.	$m_{1}^{\Omega_{22}}\left(.\right)$	$m_{2}^{\Omega_{22}}\left(.\right)$	$m_{3}^{\Omega_{22}}\left(.\right)$
$\omega_{21}≜\theta_{4}$	$\frac{3}{4}$	$\frac{1}{5}$	1
$\omega_{22}≜\theta_{5}$	$\frac{1}{4}$	$\frac{4}{5}$	0

**Table 5:** The BBAs of elements of the sub-frames Ω_3_ for Example 1.

Focal elem.	$m_{1}^{\Omega_{3}}\left(.\right)$	$m_{2}^{\Omega_{3}}\left(.\right)$	$m_{3}^{\Omega_{3}}\left(.\right)$
$\omega_{111}≜\theta_{1}$	$\frac{1}{3}$	1	0
$\omega_{112}≜\theta_{2}$	$\frac{2}{3}$	0	1

**Step 3**: The connection weights λ_*i*_ are computed from the assignments of coarsening elements. In each layer *l*, we fuse sequentially the three BBAs using PCR6 formula Eq (2). Because PCR6 fusion is not associative, we should apply the general PCR6 formula to get best results. Here we use sequential fusion to reduce the computational complexity even if the fusion result is approximate. More precisely, we compute at first $m_{12}^{PCR6,\Omega_{l}}\left(.\right)=PCR6\left(m_{1}^{\Omega_{l}}\left(.\right),m_{2}^{\Omega_{l}}\left(.\right)\right)$ and then $m_{(12)3}^{PCR6,\Omega_{l}}\left(.\right)=PCR6\left(m_{12}^{PCR6,\Omega_{l}}\left(.\right),m_{3}^{\Omega_{l}}\left(.\right)\right)$. Hence, we obtain the following connecting weights in the bintree:

- At layer *l* = 1:
  $$\begin{array}{ccc} \lambda_{1} & = & m_{(12)3}^{PCR6,\Omega_{1}}\left(\omega_{1}\right)=0.6297\\ \lambda_{2} & = & m_{(12)3}^{PCR6,\Omega_{1}}\left(\omega_{2}\right)=0.3703 \end{array}$$
- At layer *l* = 2:
  $$\begin{array}{ccc} \lambda_{3} & = & m_{(12)3}^{PCR6,\Omega_{21}}\left(\omega_{11}\right)=0.4137\\ \lambda_{4} & = & m_{(12)3}^{PCR6,\Omega_{21}}\left(\omega_{12}\right)=0.5863\\ \lambda_{5} & = & m_{(12)3}^{PCR6,\Omega_{22}}\left(\omega_{21}\right)=0.8121\\ \lambda_{6} & = & m_{(12)3}^{PCR6,\Omega_{22}}\left(\omega_{22}\right)=0.1879 \end{array}$$
- At layer *l* = 3:
  $$\begin{array}{ccc} \lambda_{7} & = & m_{(12)3}^{PCR6,\Omega_{3}}\left(\omega_{111}\right)=0.3103\\ \lambda_{8} & = & m_{(12)3}^{PCR6,\Omega_{3}}\left(\omega_{112}\right)=0.6897 \end{array}$$

**Step 4**: The final assignments of elements in original FoD Θ are calculated using the product of the connection weights of link paths from root (top) node to terminal nodes (leaves). We eventually get the *combined and normalized* Bayesian BBA:

$$\begin{array}{ccc} m^{\Theta }\left(\theta_{1}\right) & = & \lambda_{1}\cdot \lambda_{3}\cdot \lambda_{7}=0.6297\cdot 0.4137\cdot 0.3103=0.0808\\ m^{\Theta }\left(\theta_{2}\right) & = & \lambda_{1}\cdot \lambda_{3}\cdot \lambda_{8}=0.6297\cdot 0.4137\cdot 0.6897=0.1797\\ m^{\Theta }\left(\theta_{3}\right) & = & \lambda_{1}\cdot \lambda_{4}=0.6297\cdot 0.5863=0.3692\\ m^{\Theta }\left(\theta_{4}\right) & = & \lambda_{2}\cdot \lambda_{5}=0.3703\cdot 0.8121=0.3007\\ m^{\Theta }\left(\theta_{5}\right) & = & \lambda_{2}\cdot \lambda_{6}=0.3703\cdot 0.1879=0.0696 \end{array}$$

### Modified rigid coarsening

One of the issues with RC described in the previous section is that *no extra self-information of focal elements is embedded into the coarsening process*. In this paper, the elements *θ*_*i*_ selected to belong to the same group are determined using the consensus information drawn from the BBAs provided by the sources. Specifically, the degrees of disagreement between the provided sources on decisions (*θ*_1_, *θ*_2_, ⋯, *θ*_*n*_) are first calculated using the belief-interval based distance *d*_*BI*_ [20] to obtain *disagreement vector*. And then all focal elements in FoD are sorted in an ascending order. Finally, the simple dichotomous approach is utilized to hierarchical coarsen those **Re-sorted** focal elements.

#### Calculating the disagreement vector

Let us consider several BBAs $m_{s}^{\Theta }\left(\cdot \right)$, (*s* = 1, …, *S*) defined on same FoD Θ of cardinality |Θ| = *n*. The specific BBAs *m*_*θ*_*i*__(.), *i* = 1, …, *n* entirely focused on *θ*_*i*_ are defined by *m*_*θ*_*i*__(*θ*_*i*_) = 1, and for *X* ≠ *θ*_*i*_ *m*_*θ*_*i*__(*X*) = 0.

**Definition 2**: *The disagreement of opinions of two sources about* *θ*_*i*_ *is defined as the* *L*_1_-*distance between the* *d*_*BI*_ *distances of the BBAs* $m_{s}^{\Theta }\left(.\right)$, *s* = 1, 2 to *m*_*θ*_*i*__(.), *which is expressed by*

$$\begin{array}{c} D_{12}\left(\theta_{i}\right)≜\left|d_{BI}\left(m_{1}^{\Theta }\left(\cdot \right),m_{\theta_{i}}\left(\cdot \right)\right)-d_{BI}\left(m_{2}^{\Theta }\left(\cdot \right),m_{\theta_{i}}\left(\cdot \right)\right)\right| \end{array}$$ (8)

**Definition 3**: *The disagreement of opinions of* *S* ≥ 3 *sources about* *θ*_*i*_, *is defined as*

$$\begin{array}{c} D_{1-S}\left(\theta_{i}\right)≜\frac{1}{2}\sum_{i=1}^{S}\sum_{j=1}^{S}\left|d_{BI}\left(m_{i}^{\Theta }\left(\cdot \right),m_{\theta_{i}}\left(.\right)\right)-d_{BI}\left(m_{j}^{\Theta }\left(\cdot \right),m_{\theta_{i}}\left(.\right)\right)\right| \end{array}$$ (9)

*where* *d*_*BI*_ *distance is defined by* [20] *and proof of Definition 3 is given in* S1 Appendix. *For simplicity, we assume Shafer’s model so that* |2^Θ^| = 2^*n*^, *otherwise the number of elements in the summation of* Eq (10) *should be* |*D*^Θ^| − 1 *with another normalization constant* *n*_*c*_.

$$\begin{array}{c} d_{BI}^{E}\left(m_{1},m_{2}\right)≜\sqrt{n_{c}\cdot \sum_{i=1}^{2^{n}-1}{\left[d^{I}\left(BI_{1}\left(\theta_{i}\right),BI_{2}\left(\theta_{i}\right)\right)\right]}^{2}} \end{array}$$ (10)

*Here*, *n*_*c*_ = 1/2^*n*−1^ *is the normalization constant and* *d*^*I*^([*a*, *b*], [*c*, *d*]) *is the Wasserstein’s distance defined by* $d^{I}\left(\left[a,b\right],\left[c,d\right]\right)=\sqrt{{\left[\frac{a+b}{2}-\frac{c+d}{2}\right]}^{2}+\frac{1}{3}{\left[\frac{b-a}{2}-\frac{d-c}{2}\right]}^{2}}$. *And* *BI*(*θ*_*i*_) = [*Bel*(*θ*_*i*_), *Pl*(*θ*_*i*_)].

The disagreement vector **D**_1−*S*_ is defined by

$$\begin{array}{c} D_{1-S}≜\left[D_{1-S}\left(\theta_{1}\right),\ldots ,D_{1-S}\left(\theta_{n}\right)\right] \end{array}$$ (11)

#### Modified rigid coarsening by using the disagreement vector

Once *D*_1−*S*_ is derived, all focal elements {*θ*_1_, *θ*_2_, ⋯, *θ*_*n*_} are sorted according to their corresponding values in *D*_1−*S*_.

Let us revisit example 1 presented in the previous section. It can be verified in applying formula Eq (9) that the disagreement vector **D**_1−3_ for this example is equal to

$$\begin{array}{c} D_{1-3}=\left[0.4085,0.2156,0.3753,0.2507,0.4086\right] \end{array}$$

The derivation of *D*_1−3_(*θ*_1_) is given below for convenience.

$$\begin{array}{ccc} D_{1-3}\left(\theta_{1}\right) & = & |d_{BI}\left(m_{1}^{\Theta }\left(\cdot \right),m_{\theta_{1}}\left(\theta_{1}\right)\right)-d_{BI}\left(m_{2}^{\Theta }\left(\cdot \right),m_{\theta_{1}}\left(\theta_{1}\right)\right)|\\ & & +|d_{BI}\left(m_{2}^{\Theta }\left(\cdot \right),m_{\theta_{1}}\left(\theta_{1}\right)\right)-d_{BI}\left(m_{3}^{\Theta }\left(\cdot \right),m_{\theta_{1}}\left(\theta_{1}\right)\right)|\\ & & +|d_{BI}\left(m_{1}^{\Theta }\left(\cdot \right),m_{\theta_{1}}\left(\theta_{1}\right)\right)-d_{BI}\left(m_{3}^{\Theta }\left(\cdot \right),m_{\theta_{1}}\left(\theta_{1}\right)\right)|\\ & = & 0.4085. \end{array}$$

**Fig 2:**
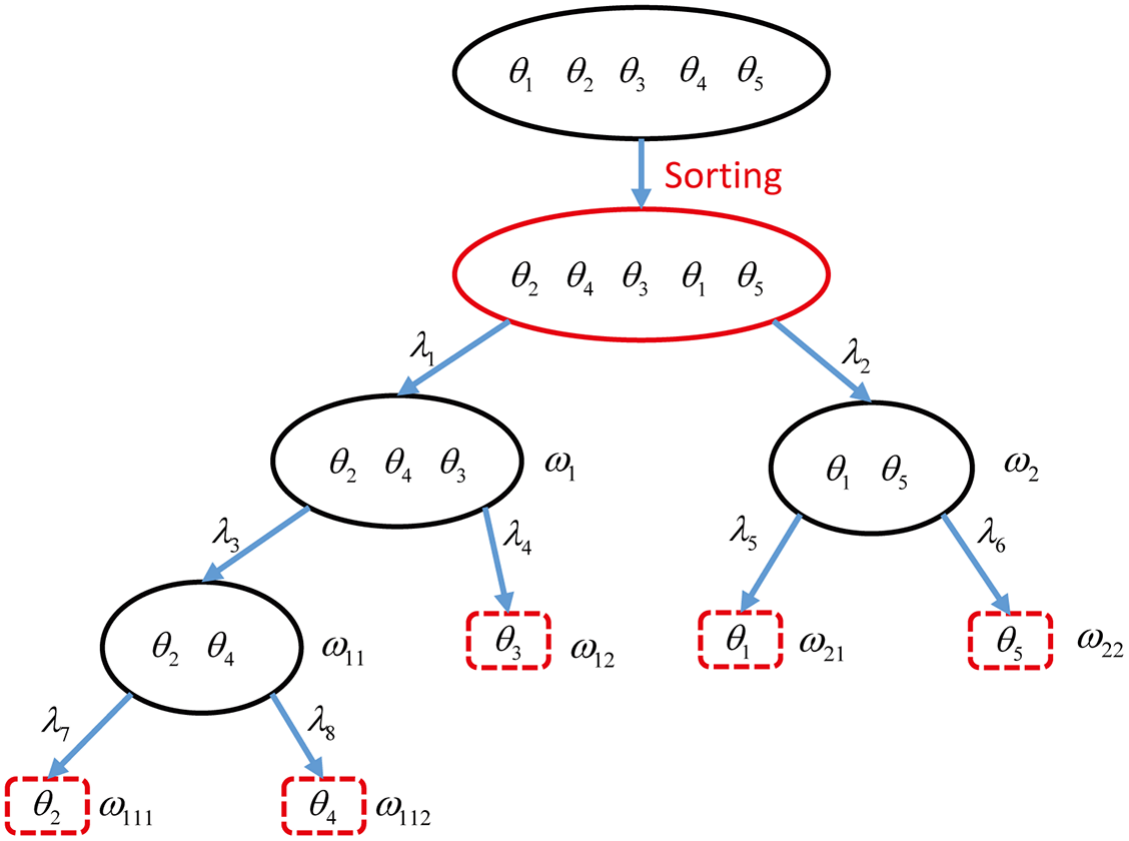
Fusion of Bayesian BBAs using MRC for Example 1.

Based on the disagreement vector, a new bintree structure is obtained and shown in Fig 2. Compared with Fig 1, the elements in FoD Θ are grouped more reasonably. In vector **D**_1−3_, *θ*_1_ and *θ*_5_ lie in similar degree of disagreement so that they are put in the same group. Similarly for *θ*_2_ and *θ*_4_. However, element *θ*_3_ seems *weird*, which is put alone in the process of coarsening. Once this new bintree decomposition is obtained, other steps can be implemented which are identical to rigid coarsening in section to get the final combined BBA.

**Step 1**: According to Fig 2, the elements of the frames Ω_*l*_ are defined as follows:

- At layer *l* = 1: $\Omega_{1}=\left\{\omega_{1}≜\theta_{2}\cup \theta_{4}\cup \theta_{3},\omega_{2}≜\theta_{1}\cup \theta_{5}\right\}$
- At layer *l* = 2: $\Omega_{2}=\left\{\omega_{11}≜\theta_{2}\cup \theta_{4},\omega_{12}≜\theta_{3},\omega_{21}≜\theta_{1},\omega_{22}≜\theta_{5}\right\}$
- At layer *l* = 3: $\Omega_{3}=\left\{\omega_{111}≜\theta_{2},\omega_{112}≜\theta_{4}\right\}$

**Step 2**: The BBAs of elements of the (sub-) frames Ω_*l*_ are obtained as follows:

- At layer *l* = 1, we use Eqs (6) and (7) and we get (Table 6)
- At layer *l* = 2: We use again the proportional redistribution method which gives us Tables 7 and 8. Here, masses of *ω*_21_, *ω*_22_ in $m_{3}^{\Omega_{22}}\left(.\right)$ are not considered because the mass of their parent focal element ($m_{3}^{\Omega_{1}}\left(\omega_{2}\right)$) in bintree is 0.
- At layer *l* = 3: We work with the two subframes $\Omega_{3}≜\left\{\omega_{111},\omega_{112}\right\}$ of Ω_3_ with the BBAs in Table 9:

**Table 6:** The BBAs of elements of the sub-frames Ω_1_ Using MRC for Example 1.

Focal elem.	$m_{1}^{\Omega_{1}}\left(.\right)$	$m_{2}^{\Omega_{1}}\left(.\right)$	$m_{3}^{\Omega_{1}}\left(.\right)$
$\omega_{1}≜\theta_{2}\cup \theta_{4}\cup \theta_{3}$	0.8	0.2	1.0
$\omega_{2}≜\theta_{1}\cup \theta_{5}$	0.2	0.8	0.0

**Table 7:** The BBAs of elements of the sub-frames Ω_21_ Using MRC for Example 1.

Focal elem.	$m_{1}^{\Omega_{21}}\left(.\right)$	$m_{2}^{\Omega_{21}}\left(.\right)$	$m_{3}^{\Omega_{21}}\left(.\right)$
$\omega_{11}≜\theta_{2}\cup \theta_{4}$	$\frac{5}{8}$	$\frac{1}{2}$	$\frac{1}{2}$
$\omega_{12}≜\theta_{3}$	$\frac{3}{8}$	$\frac{1}{2}$	$\frac{1}{2}$

**Table 8:** The BBAs of elements of the sub-frames Ω_22_ Using MRC for Example 1.

Focal elem.	$m_{1}^{\Omega_{22}}\left(.\right)$	$m_{2}^{\Omega_{22}}\left(.\right)$	$m_{3}^{\Omega_{22}}\left(.\right)$
$\omega_{21}≜\theta_{1}$	$\frac{1}{2}$	$\frac{1}{2}$	–
$\omega_{22}≜\theta_{5}$	$\frac{1}{2}$	$\frac{1}{2}$	–

**Table 9:** The BBAs of elements of the sub-frames Ω_3_ Using MRC for Example 1.

Focal elem.	$m_{1}^{\Omega_{3}}\left(.\right)$	$m_{2}^{\Omega_{3}}\left(.\right)$	$m_{3}^{\Omega_{3}}\left(.\right)$
$\omega_{111}≜\theta_{2}$	$\frac{2}{5}$	0.0	$\frac{1}{5}$
$\omega_{112}≜\theta_{4}$	$\frac{3}{5}$	1.0	$\frac{4}{5}$

**Step 3**: The connection weights λ_*i*_ are computed from the assignments of coarsening elements. Hence, we obtain the following connecting weights in the bintree:

- At layer *l* = 1:
  $$\begin{array}{c} \lambda_{1}=0.8333;\;\;\lambda_{2}=0.1667. \end{array}$$
- At layer *l* = 2:
  $$\begin{array}{c} \lambda_{3}=0.5697;\;\;\lambda_{4}=0.4303;\\ \lambda_{5}=0.5000;\;\;\lambda_{6}=0.5000. \end{array}$$
- At layer *l* = 3:
  $$\begin{array}{c} \lambda_{7}=0.0669;\;\;\lambda_{8}=0.9331; \end{array}$$

**Step 4**: We finally get the following *combined and normalized* Bayesian BBA

$$\begin{array}{c} m^{\Theta }\left(\cdot \right)=\left\{0.0833,0.0318,0.3586,0.4430,0.0834\right\}. \end{array}$$

### Summary of the proposed method

**Fig 3:**
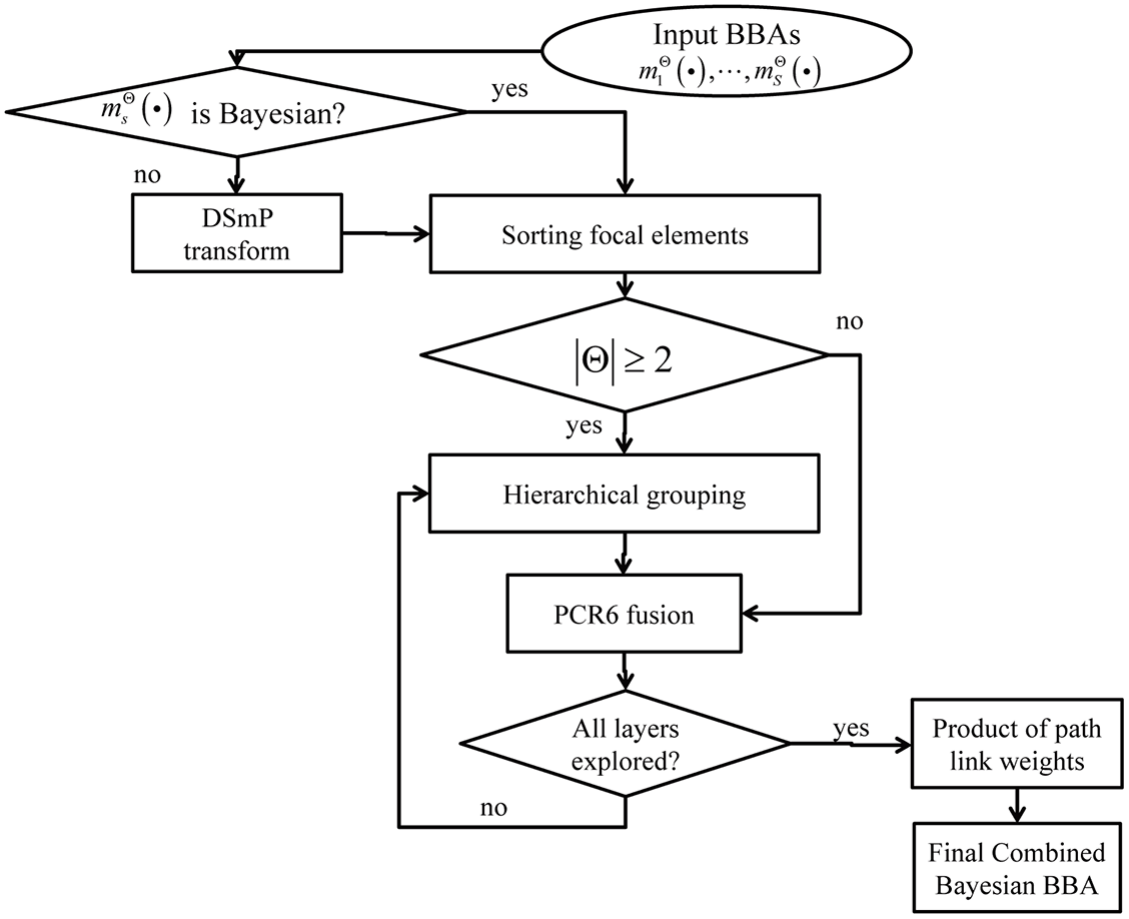
Modified rigid coarsening of FoD for fusion.

The fusion method of BBAs to get a combined Bayesian BBA based on hierarchical decomposition of the FoD consists of several steps (**Algorithm 1**) below illustrated in Fig 3. It is worth noting that when the given BBAs are not Bayesian, the first step is to use the existing Probabilistic Transformation (PT) to transform them to Bayesian BBAs. In order to use the proposed combination method in the RSs, modified rigid coarsening is mathematically denoted as ⨁ in the following sections.

**Algorithm 1**: Modified Rigid Coarsening Method

**Input:** All original BBAs $m_{1}^{\Theta }\left(\cdot \right),\cdots ,m_{s}^{\Theta }\left(\cdot \right)$, *s* = 1, 2, ⋯, *s*

**Output:** The final combined BBA *m*^Θ^(⋅)

1 **if** Compound focal elements in Θ: *θ*_*i*_ ∪ *θ*_*j*_ ≠ ∅ or *θ*_*i*_ ∩ *θ*_*j*_ ≠ ∅ **then**

2  Probabilistic transformation: $DSmP\left(m_{1}^{\Theta }\left(\cdot \right)\right),DSmP\left(m_{2}^{\Theta }\left(\cdot \right)\right),\cdots ,DSmP\left(m_{s}^{\Theta }\left(\cdot \right)\right)$

3 **end**

4 **for** *i* ≤ *n* **do**

5  **for** *s* ≤ *S* **do**

6   Calculate $D_{1-S}\left(\theta_{i}\right)≜\frac{1}{2}\sum_{i=1}^{S}\sum_{j=1}^{S}\left|d_{BI}\left(m_{i}^{\Theta }\left(\cdot \right),m_{\theta_{i}}\left(.\right)\right)-d_{BI}\left(m_{j}^{\Theta }\left(\cdot \right),m_{\theta_{i}}\left(.\right)\right)\right|$

7  **end**

8 **end**

9 **for** *i* ≤ *n* **do**

10  Sorting *D*_1−*S*_(*θ*_*i*_) in an ascending order.

11 **end**

12 **while** |Θ| ≥ 2 **do**

13  **if** *n is an even number* **then**

14   $m^{\Omega_{l}}\left(\omega_{1}\right)=\sum_{j=1,\ldots ,\frac{n}{2}}m^{\Theta }\left(\theta_{j}\right)$;

15   $m^{\Omega_{l}}\left(\omega_{2}\right)=\sum_{j=\frac{n}{2}+1,\ldots ,n}m^{\Theta }\left(\theta_{j}\right)$;

16  **else**

17   $m^{\Omega_{l}}\left(\omega_{1}\right)=\sum_{j=1,\ldots ,[\frac{n}{2}+1]}m^{\Theta }\left(\theta_{j}\right)$;

18   $m^{\Omega_{l}}\left(\omega_{2}\right)=\sum_{j=[\frac{n}{2}+1]+1,\ldots ,n}m^{\Theta }\left(\theta_{j}\right)$;

19  **end**

20  Then connection weights λ is calculated: *PCR*6(*m*^Ω^(*ω*_1_), *m*^Ω^(*ω*_2_))

21 **end**

22 **foreach** focal element *θ*_*i*_, *i* ∈ 1, ⋯, *n* **do**

23  *m*^Θ^(*θ*_*i*_) equals to the product of path link weights from root to terminal nodes.

24 **end**

### Simulation considering accuracy and computational efficiency

**Fig 4:**
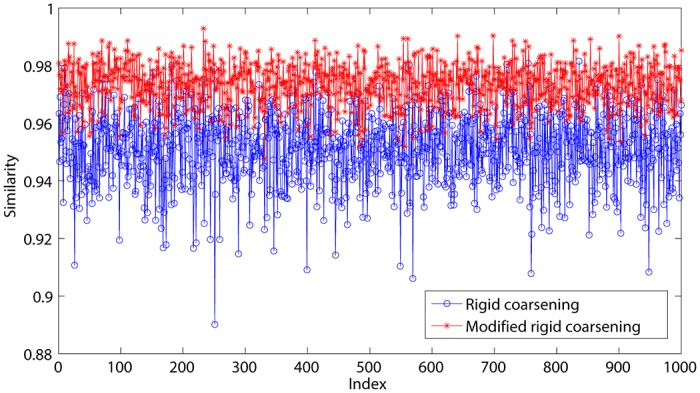
Accuracy comparisons between MRC and PCR6 (Only Singletons).

**Fig 5:**
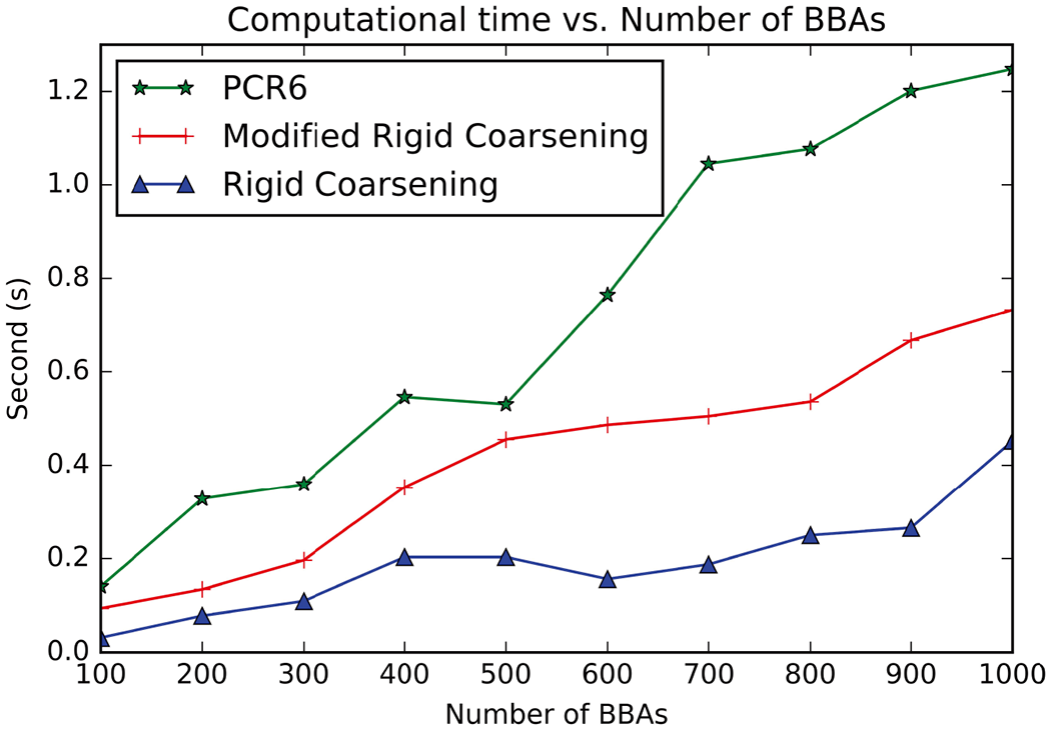
Efficiency comparisons between MRC, RC and PCR6 (With the number of BBAs increasing).

**Fig 6:**
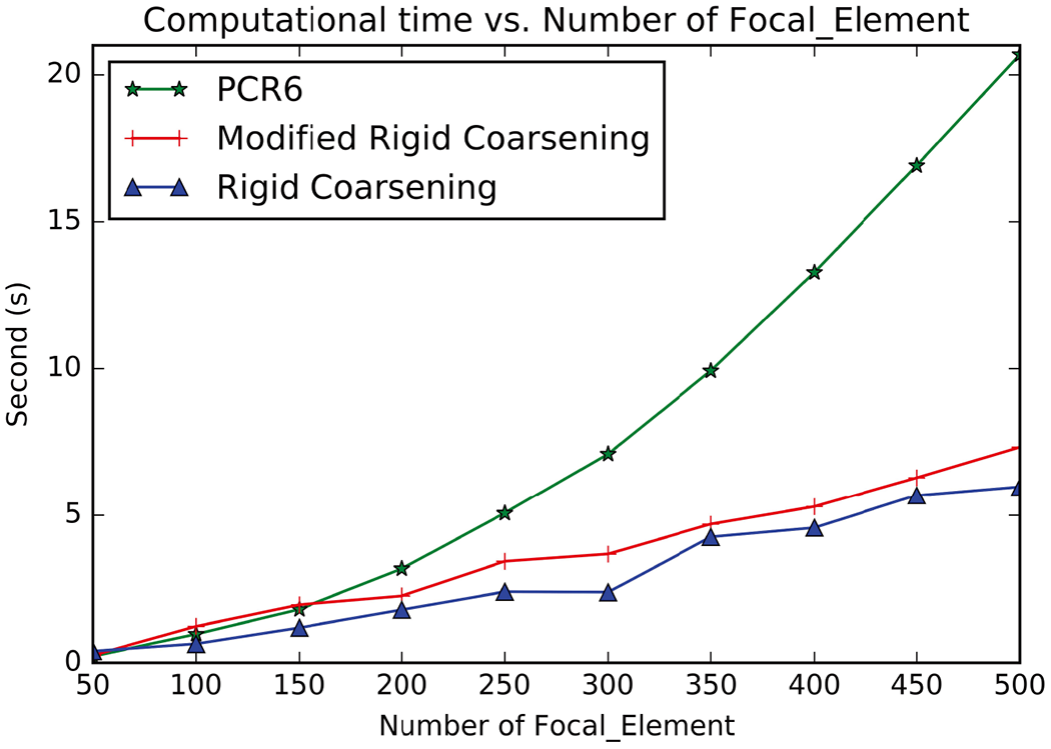
Efficiency comparisons between MRC, RC and PCR6 (With the number of focal elements increasing).

- Accuracy:
  Assuming that the FoD is Θ = {*θ*_1_, *θ*_2_, *θ*_3_, *θ*_4_, *θ*_5_, *θ*_6_, *θ*_7_, *θ*_8_, *θ*_9_, *θ*_10_, *θ*_11_, *θ*_12_, *θ*_13_, *θ*_14_, *θ*_15_, *θ*_16_, *θ*_17_, *θ*_18_, *θ*_19_, *θ*_20_}, then 1000 BBAs are randomly generated to be fused with three methods: modified rigid coarsening, rigid coarsening and also PCR6. And then distances of fusion results are computed using *d*_*BI*_ between two pairs: modified rigid coarsening and PCR6; rigid coarsening and PCR6. Comparisons are made in Fig 4, which show the superiority of our new approach proposed in this paper (The average value of the approximation of modified rigid coarsening is **97.5%** and original rigid coarsening is **94.5%**). Here, similarity represents the approximate degree between fusion results using hierarchical approximate method (both rigid and modified rigid coarsening) and PCR6.
- Computational efficiency:
  As we mentioned before, another advantage of the hierarchical combination method is the computational efficiency. Here, two experiments are conducted (*All experiments are implemented on a PC with I3 CPU, Integrated graphics chipsets and 4G DDR*): 1) the number of singletons is unchanged while the number of BBAs to be fused is increasing; 2) the number of BBAs is unchanged while the number of singletons in FoD is increasing. The results are illustrated in Figs 5 and 6. From experiment 1, all these three methods (classical PCR6, rigid coarsening and also modified rigid coarsening) calculate quickly (**less than 1.2s**) even the number of BBAs increases from 100 to 1000. However, such situation deteriorates when the number of focal elements increases. In Fig 6, when the number of focal elements increases to 500, time consumption of three combinations is: PCR6: 20.6857s; modified rigid coarsening: 7.3320s; rigid coarsening: 5.9748s. This phenomenon also proves that it is reasonable to map original FoD to the coarsening FoD, with the aim of reducing the number of focal elements at the time of fusion. But in any case, computing efficiency of rigid coarsening or modified rigid coarsening is still better than PCR6. On the other hand, modified rigid coarsening makes a significant improvement (accuracy) at the expense of parts of the computational efficiency.

## A recommender system integrating with hierarchical coarsening combination method

In today’s e-commerce, online providers often recommend proper goods or services to each consumer based on their personal opinions or preferences [21], [22]. However, it is a tough task to provide appropriate recommendation which may confront several difficulties. One difficulty is that users’ preferences are usually characterized as uncertain, imprecise or incomplete [23], [24], which cannot be used directly in RSs. Besides, it is easy to understand that when the more information about user preferences are, the more accurate prediction of RSs will be [25], [26]. But, the problem is that which method we adopt to integrate multi-source uncertain information?

As a general framework for information fusion, DST can not only model uncertain information, but also provide an efficient way to combine multi-source information. These mentioned features make this theory a wide range of applications [27–29], especially in RSs [23, 25, 30–32]. According to DST, users’ comments on products in RSs are described by using mass functions and rules of combination method are used frequently in order to provide appropriate recommendation.

As mentioned in previous sections, both the performances of combination rules in DST or in DSmT suffer from computational complex which is obviously ignored in [23, 25]. Thus, in this paper, modified rigid coarsening method is applicable to combine the imprecise users’ preferences in RSs. First, we are required to introduce the relevant knowledge of RSs. Actually, almost all characteristics of RSs have been introduced in [23, 25, 30–32].

First, we give the corresponding representation of the mathematical notation in RSs based on DSmT. RSs usually contain two objects: {*Users*, *Items*}. A set of *M* users and a set containing *N* items is respectively denoted by **U** = {*U*_1_, *U*_2_, ⋯, *U*_*M*_} and **I** = {*I*_1_, *I*_2_, ⋯, *I*_*N*_}. Besides, we assume that users can give the corresponding ratings to the items, which include *L* rating levels (Θ = {*θ*_1_, *θ*_2_, ⋯, *θ*_*L*_}.). Here, *L* preference levels means multi-level evaluation results. For example, four-levels of user evaluation on the product are {*Excellent*, *Good*, *Fair*, *Poor*}. *r*_*i*,*k*_ means a rating of user *U*_*i*_ on item *I*_*k*_ and a rating matrix **R** = {*r*_*i*,*k*_} comprises all the ratings of users on items. It should be noted that *r*_*i*,*k*_ is originally modeled as a mass function *m*_*i*,*k*_: *D*^Θ^ → [0, 1]. Additionally, let $I_{i}^{R}$ and $U_{k}^{R}$ denote the set of items rated by user *U*_*i*_ and the set of users having rated item *I*_*k*_, respectively.

**Fig 7:**
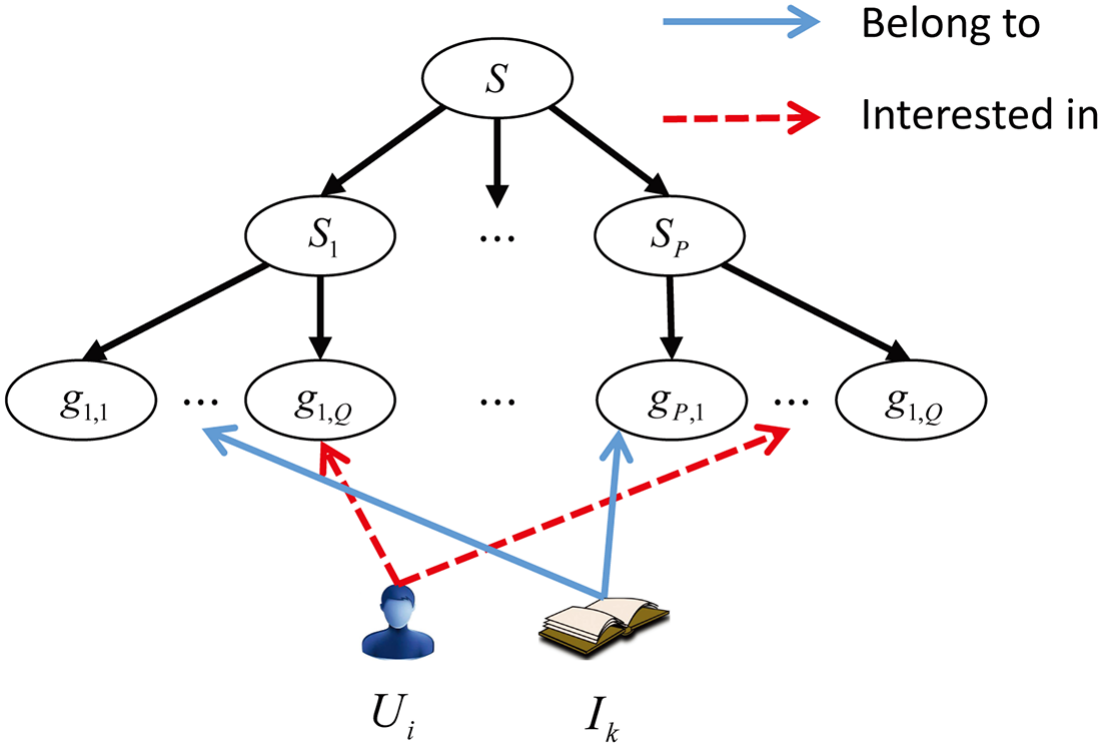
Contextual information.

Contextual information can often be summarized into several genres that significantly affect user’s rating of items. Normally, we represent contextual information by a set containing *P* genres, denoted by **S** = {*S*_1_, *S*_2_, ⋯, *S*_*P*_}. And each genre *S*_*p*_, with 1 ≤ *p* ≤ *P* contains at most *Q* groups, denoted by *S*_*p*_ = {*g*_*p*,1_, *g*_*p*,2_, ⋯, *g*_*p*,*q*_, ⋯, *g*_*p*,*Q*_}, 1 ≤ *q* ≤ *Q*. For a genre *S*_*p*_ ∈ **S**, a user *U*_*i*_ ∈ **U** can be interested in several groups and also an item *I*_*i*_ ∈ **I** can belong to one or some groups of this genre, which can be seen in Fig 7.

**Definition 4**: *In order to facilitate such expression, two functions* *κ*(⋅) *and* *φ*(⋅) *are defined to determine the groups in which user* *U*_*i*_ *is interested and the groups to which item* *I*_*k*_ *belongs, respectively*:

$$\begin{array}{c} \kappa_{p}:U_{i}\mapsto \kappa_{p}\left(U_{i}\right)\subseteq S_{p} \end{array}$$ (12)

$$\begin{array}{c} \varphi_{p}:I_{k}\mapsto \varphi_{p}\left(I_{k}\right)\subseteq S_{p} \end{array}$$ (13)

**Fig 8:**
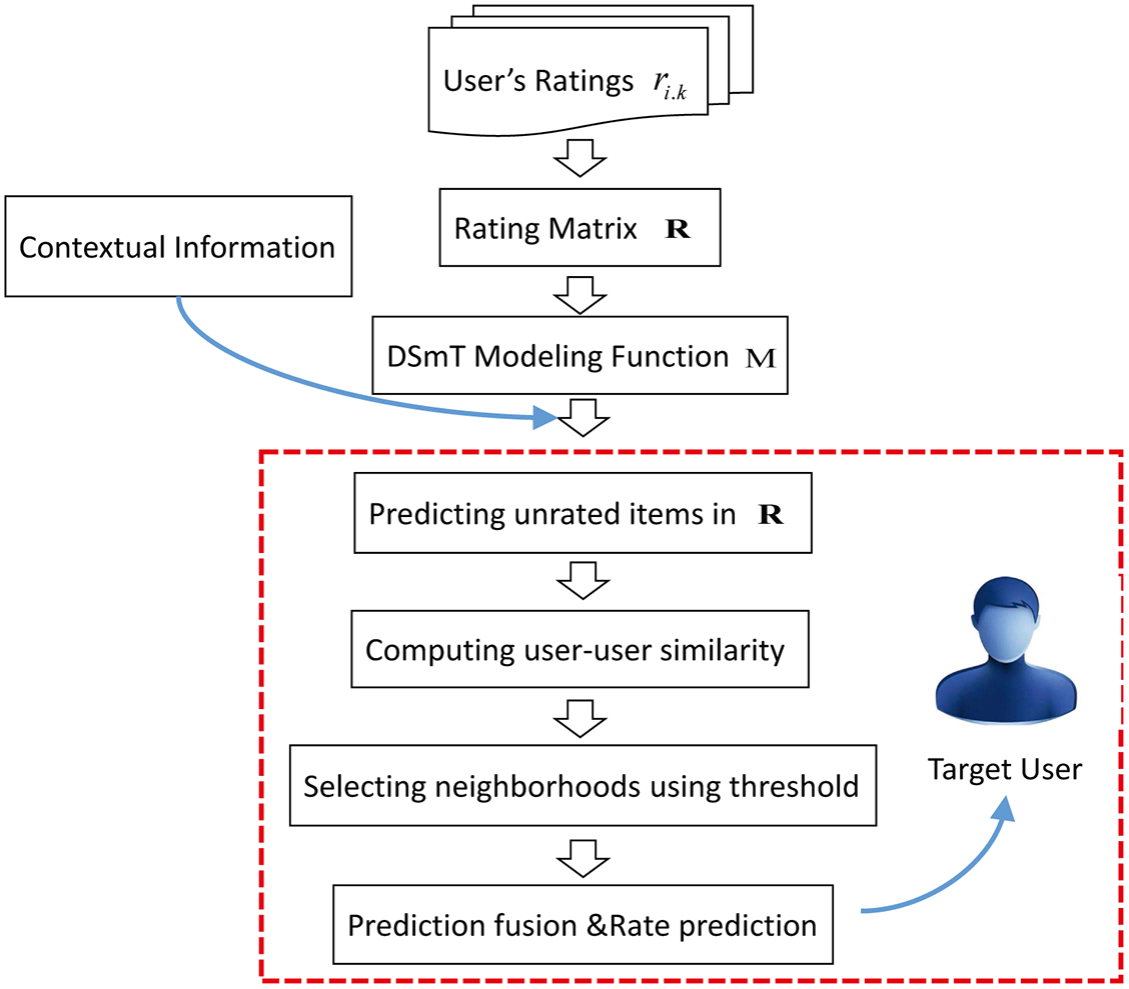
General process of recommendations.

**Fig 9:**
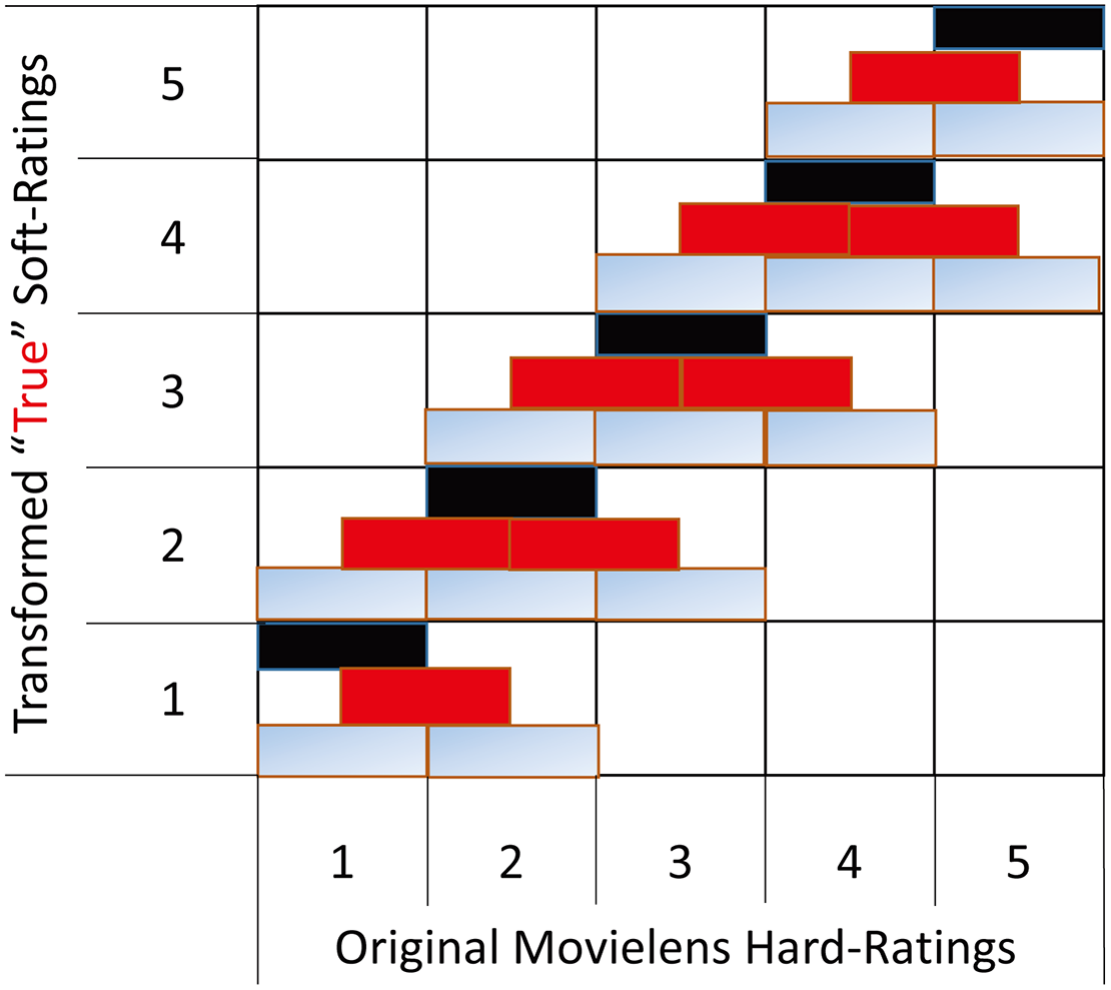
DSmT modeling function.

Generally, the main steps of a recommendation system is illustrated in Fig 8, which will be presented in details as follows:

1. DSmT-Modeling Function
  Regarding the DS-partial probability models proposed in [23], the existing ratings *r*_*i*,*k*_, of user *U*_*i*_ on item *I*_*k*_, are modeled by DSmT-modeling function *M*(⋅) in order to transform such hard ratings into the corresponding soft ratings represented as *m*_*i*,*k*_ as below:
  **Definition 5**:
  $$\begin{array}{c} m_{i,k}=\left\{ \begin{array}{cc} \alpha_{i,k}\left(1-\sigma_{i,k}\right), & \;\;for\;\;A=\theta_{l};\\ \frac{1}{2}\alpha_{i,k}\sigma_{i,k}, & \;\;for\;\;A=B;\\ \frac{1}{2}\alpha_{i,k}\sigma_{i,k}, & \;\;for\;\;A=C;\\ 1-\alpha_{i,k}, & \;\;for\;\;A=\Theta ;\\ 0, & \;\;otherwise. \end{array}\right. \end{array}$$ (14)
  *with*
  $$B=\{\begin{array}{cc} \theta_{1}\cup \theta_{2}, & \;\;if\;\;l=1;\\ \theta_{L-1}\cup \theta_{L}, & \;\;if\;\;l=L;\\ \theta_{l-1}\cup \theta_{l}\cup \theta_{l+1}, & \;\;otherwise. \end{array}$$
  $$C=\{\begin{array}{cc} \theta_{1}\cap \theta_{2}, & \;\;if\;\;l=1;\\ \theta_{L-1}\cap \theta_{L}, & \;\;if\;\;l=L;\\ (\theta_{l-1}\cap \theta_{l},\theta_{l}\cap \theta_{l+1}), & \;\;otherwise. \end{array}$$
  *where* *α*_*i*,*k*_ ∈ [0, 1] *and* *σ*_*i*,*k*_ *are a trust factor and a dispersion factor, respectively* [23].
  Referring to the partial probability model analysis in [23], we also give the corresponding user profiles which can be seen in Fig 9. Compared to [23], the difference is that we not only consider the union (*black and gray rectangle*), but also consider the intersection (*red rectangle*) of the hard ratings, which is also the distinction between DS theory and DSmT theory.
  **Lemma 1:** Referring to Definition 5, we can also generate the relative refined BBA in the framework of DS theory:
  $$\begin{array}{c} m_{i,k}^{Refined}=\left\{ \begin{array}{cc} \alpha_{i,k}\left(1-\sigma_{i,k}\right), & \;\;for\;\;A=\theta_{l};\\ \alpha_{i,k}\sigma_{i,k}, & \;\;for\;\;A=B;\\ 1-\alpha_{i,k}, & \;\;for\;\;A=\Theta ;\\ 0, & \;\;otherwise. \end{array}\right. \end{array}$$ (15)
  with
  $$B=\{\begin{array}{cc} \theta_{1}\cup \theta_{2}, & \;\;if\;\;l=1;\\ \theta_{L-1}\cup \theta_{L}, & \;\;if\;\;l=L;\\ \theta_{l-1}\cup \theta_{l}\cup \theta_{l+1}, & \;\;otherwise. \end{array}$$
  where *α*_*i*,*k*_ ∈ [0, 1] and *σ*_*i*,*k*_ are a trust factor and a dispersion factor, respectively [23].
  After soft ratings are generated, DSmP [33] is applied to decouple non-Bayesian *m*_*i*,*k*_, since the hierarchical fusion algorithm is currently just available for Bayesian BBAs.
  **Definition 6**: *DSmP is a new generalized pignistic transformation defined by* *DSmP*_*ε*_(∅) = 0 *and for any singleton* *θ*_*i*_ ∈ Θ *by*
  $$\begin{array}{c} DSmP_{\varepsilon }\left(\theta_{i}\right)≜m\left(\theta_{i}\right)+\left(m\left(\theta_{i}\right)+\varepsilon \right)\\ \times \{\sum_{A\in 2^{\Theta },\theta_{N}\subset A,\left|A\right|\geq 2}\frac{m(A)}{\sum_{B\in 2^{\Theta },B\subset A,\left|B\right|=1}m\left(B\right)+\varepsilon \cdot \left|A\right|}\} \end{array}$$ (16)
  As shown in [33], DSmP makes a remarkable improvement compared with BetP and CuzzP, since a more judicious redistribution of the ignorance masses to the singletons has been adopted by DSmP.
2. Predicting unrated items:
  Assuming that users who are keen on the similar groups tend to have common preferences. In this RS, it is necessary to predict the unrated items first. Considering a group *g*_*p*,*q*_ ∈ *S*_*p*_ with *g*_*p*,*q*_ ∈ *φ*(*I*_*k*_), every soft rating, *m*_*i*,*k*_, of user *U*_*i*_, who is keen on group *g*_*p*,*q*_, on item *I*_*k*_ is regarded as a block of common preference for group *g*_*p*,*q*_. Thus, *G*_*m*_*p*,*q*,*k*__: *D*^Θ^ → [0, 1] which represents all users’ group preferences on item *I*_*k*_ regarding group *g*_*p*,*q*_, is computed as follows
  $$\begin{array}{c} G_{m_{p,q,k}}=\underset{\left\{j|I_{k}\in I_{j}^{R},g_{p,q}\in \kappa_{p}\left(U_{j}\right),g_{p,q}\in \varphi_{p}\left(I_{k}\right)\right\}}{⨁}m_{j,k} \end{array}$$ (17)
  Supposing that item *I*_*k*_ has not been rated by user *U*_*i*_, it usually contains three steps to generate unprovided rating *r*_*i*,*k*_ of user *U*_*i*_ which are shown as below
  - Step one: Considering a concept *S*_*p*_, for each group *g*_*p*,*q*_ ∈ *κ*_*p*_(*U*_*i*_) ∩ *φ*_*p*_(*I*_*k*_), it is assumed that all users’ group preferences on item *I*_*k*_ regarding group *g*_*p*,*q*_ imply common preference of *U*_*i*_ on *I*_*k*_ regarding group *g*_*p*,*q*_. Furthermore, this group preference is regarded as a piece of user *U*_*i*_’s concept preference on item *I*_*k*_ regarding concept *S*_*p*_. Therefore, concept preference of user *U*_*i*_ on item *I*_*k*_ regarding concept *S*_*p*_, denoted by mass function *S*_*m*_*p*,*q*,*k*__: *D*^Θ^ → [0, 1], can be computed as below
    $$\begin{array}{c} S_{m_{p,q,k}}=\underset{\left\{q|g_{p,q}\in \kappa_{p}\left(U_{i}\right),g_{p,q}\in \varphi_{p}\left(I_{k}\right)\right\}}{⨁}G_{m_{p,q,k}} \end{array}$$ (18)
  - Step two: If there exists at least one common group in concept *S*_*p*_ which item *I*_*k*_ belongs to and also user *U*_*i*_ is interested in, then *U*_*i*_’s concept preference on item *I*_*k*_ regarding concept *S*_*p*_ is regarded as a piece of context preference. Therefore, this user’s contextual preference on item *I*_*k*_, denoted by mass function *S*_*m*_*i*,*k*__: *D*^Θ^ → [0, 1], is achieved as follows
    $$\begin{array}{c} S_{m_{i,k}}=\underset{p=1,\cdots ,P}{⨁}S_{m_{p,i,k}} \end{array}$$ (19)
  - Step three: Context preference of *U*_*i*_ on item *I*_*k*_ is assigned to unprovided rating ${\bar{m}}_{i,k}$ as below
    $$\begin{array}{c} {\bar{m}}_{i,k}=S_{m_{i,k}} \end{array}$$ (20)
  So far, all unprovided ratings are predicted in this RS. Subsequently, user-user similarities are computed depending on both provided and predicted ratings in the following steps.
3. Computing user-user similarities:
  Here, we use the distance measure proposed in [34] to calculate distances between two users *U*_*i*_ and *U*_*j*_ with *i* ≠ *j*, which is defined as below
  $$\begin{array}{c} \begin{array}{cc} D(U_{i},U_{j}) & =\sum_{k=1}^{N}\left(\ln\max_{\theta \in \Theta }\frac{m_{j,k}\left(\theta \right)}{m_{i,k}\left(\theta \right)}-\ln\min_{\theta \in \Theta }\frac{m_{j,k}\left(\theta \right)}{m_{i,k}\left(\theta \right)}\right) \end{array} \end{array}$$ (21)
  where *m*_*i*,*k*_ and *m*_*j*,*k*_ are the soft ratings of user *U*_*i*_ and user *U*_*j*_ on item *I*_*k*_ respectively. Afterwards, the degree of similarity between *U*_*i*_ and *U*_*j*_, denoted by *s*_*i*,*j*_, is calculated as follows
  $$\begin{array}{c} s_{i,j}=e^{-\gamma \times D(U_{i},U_{j})},where\;\;\gamma \in \left(0,\infty \right). \end{array}$$ (22)
  Obviously, if the value of *s*_*i*,*j*_ is high, it means the user *U*_*i*_ and user *U*_*j*_ are very close, and vice versa. Eventually, a mathematical matrix **S** = {*s*_*i*,*j*_|*U*_*i*_, *U*_*j*_ ∈ **U**, *i* ≠ *j*} is employed to represent the similarities among all users.
4. Selecting neighbors based on user-user similarities:
  Taking into account an active user *U*_*i*_, for each unrated item *I*_*k*_ by user *U*_*i*_, a set containing *K* nearest neighborhoods, denoted by $N_{i,k}$, is chosen by using the method proposed in [35]. Two simple steps of this method are shown below
  - Step one: the process of such selection depends on two criteria: 1. Those users who rated *I*_*k*_ and 2. The corresponding user-user similarities with user *U*_*i*_ are equal or greater than the threshold *τ*. $N_{i,k}$ denotes the selected set, which is acquired as follows:
    $$\begin{array}{c} N_{i,k}=\left\{U_{j}\in U|I_{k}\in I_{j}^{R},s_{i,j}\geq \tau \right\} \end{array}$$ (23)
  - Step two: all of members in $N_{i,k}$ is descending sorted by *s*_*i*,*j*_ and top *K* members are selected as the neighborhood set $N_{i,k}$.
5. Estimating ratings according to neighborhoods:
  Supposing that item *I*_*k*_ has not been rated by user *U*_*i*_. The predicted rating of *U*_*i*_ on item *I*_*k*_ is denoted as ${\hat{m}}_{i,k}$. Thus, ${\hat{m}}_{i,k}$ is calculated according to the ratings of user *U*_*i*_’s nearest users. Mathematically, ${\hat{m}}_{i,k}$ is given as below
  $$\begin{array}{c} {\hat{m}}_{i,k}=m_{i,k}⨁{\tilde{m}}_{i,k} \end{array}$$ (24)
  where ${\tilde{m}}_{i,k}$ is the mass regarding the neighborhoods’ whole preference in the set Eq (23) on item *I*_*k*_. Considering user $U_{i}\in N_{i,k}$, and supposing that *s*_*i*,*j*_ is the similarity between user *U*_*i*_ and user *U*_*j*_. We use a discount rate 1 − *s*_*i*,*j*_ to discount the rating of user *U*_*j*_ on item *I*_*k*_. Therefore, ${\tilde{m}}_{i,k}$ is:
  $$\begin{array}{c} {\tilde{m}}_{i,k}=\underset{\left\{j|U_{j}\in N_{i,k}\right\}}{⨁}{\dot{m}}_{j,k}^{s_{i,j}} \end{array}$$ (25)
  $where\;\;{\dot{m}}_{j,k}^{s_{i,j}}=\{\begin{array}{cc} s_{i,j}\times m_{j,k}\left(A\right), & for\;A\subset \Theta ;\\ s_{i,j}\times m_{j,k}\left(\Theta \right)+\left(1-s_{i,j}\right), & if\;\;A=\Theta . \end{array}$
6. Generating recommendations:
  In order to generate appropriate recommendations for the candidate user *U*_*i*_, predicted ratings of *U*_*i*_ on all unprovided items are sorted, and then based on the sorted list, the appropriate recommendations are generated.

## Experiments

To evaluate the performance of modified rigid coarsening in precision of recommendation and computational time, original rigid coarsening method and also classical PCR6 combination method are selected to be regarded as baselines. Besides, we use DS-MAE [23] to measure the precision of recommendations.

**Definition 7**: *DS-MAE is mathematically given as follows*

$$\begin{array}{c} DS\text{-}MAE\left(\theta_{j}\right)=\frac{1}{|D_{j}|}\sum_{\left(i,k\right)\in D_{j},\theta_{l}\in \Theta }\left|{\hat{m}}_{i,k}\left(\theta_{j}\right)-M\left(\theta_{j}\right)\right| \end{array}$$ (26)

*where* *D*_*j*_ *is the testing set identifying the user-item pairs whose true rating is* *θ*_*j*_ ∈ Θ.

Those specific users’ interested information about genres is unknown. Thus, we define a rule that if a user has rated an item then this user is interested in all genres to which the item belongs.

**Fig 10:**
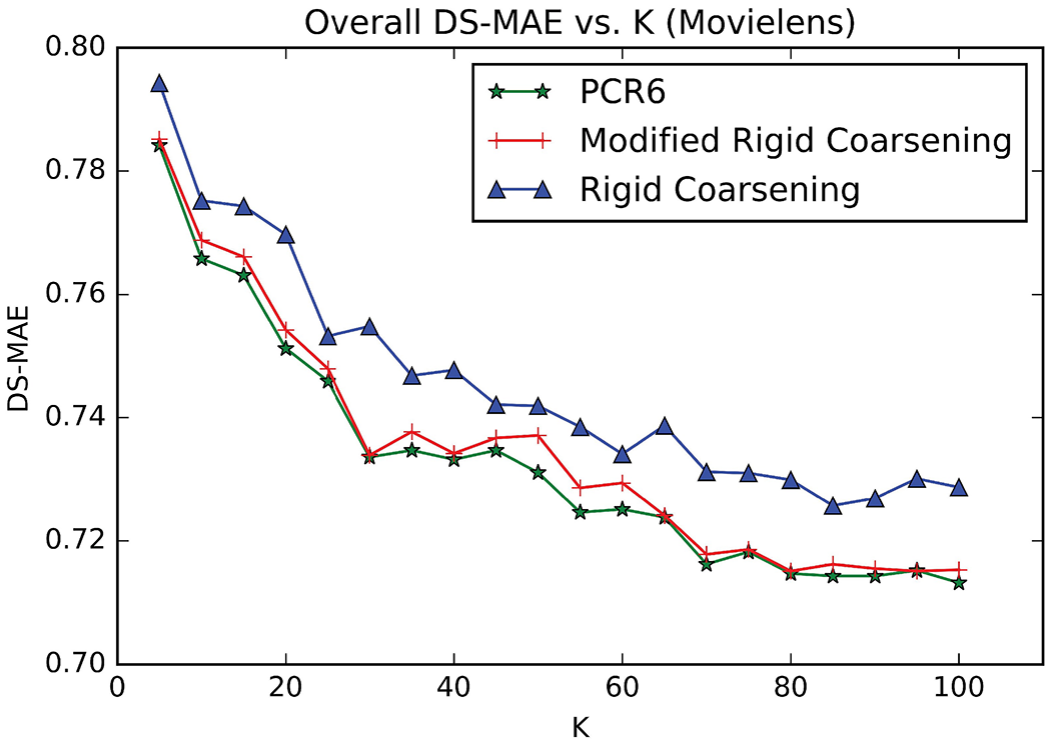
Overall DS-MAE between three combination methods. (Movielens).

**Fig 11:**
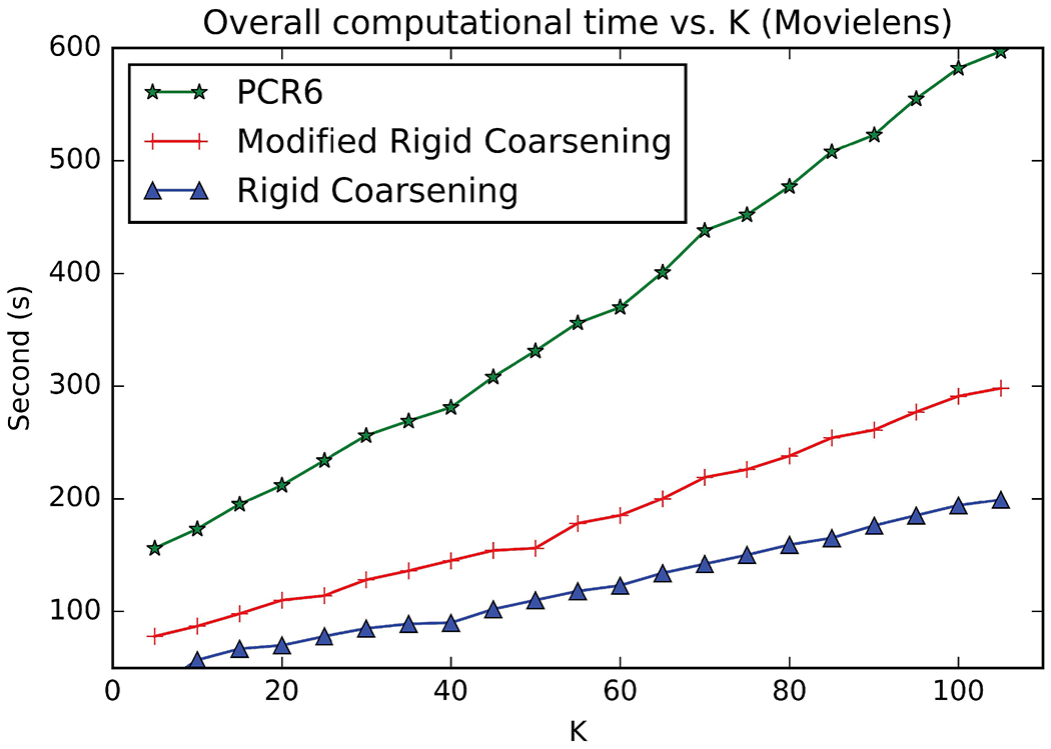
Overall computational time between three combination methods. (Movielens).

**Fig 12:**
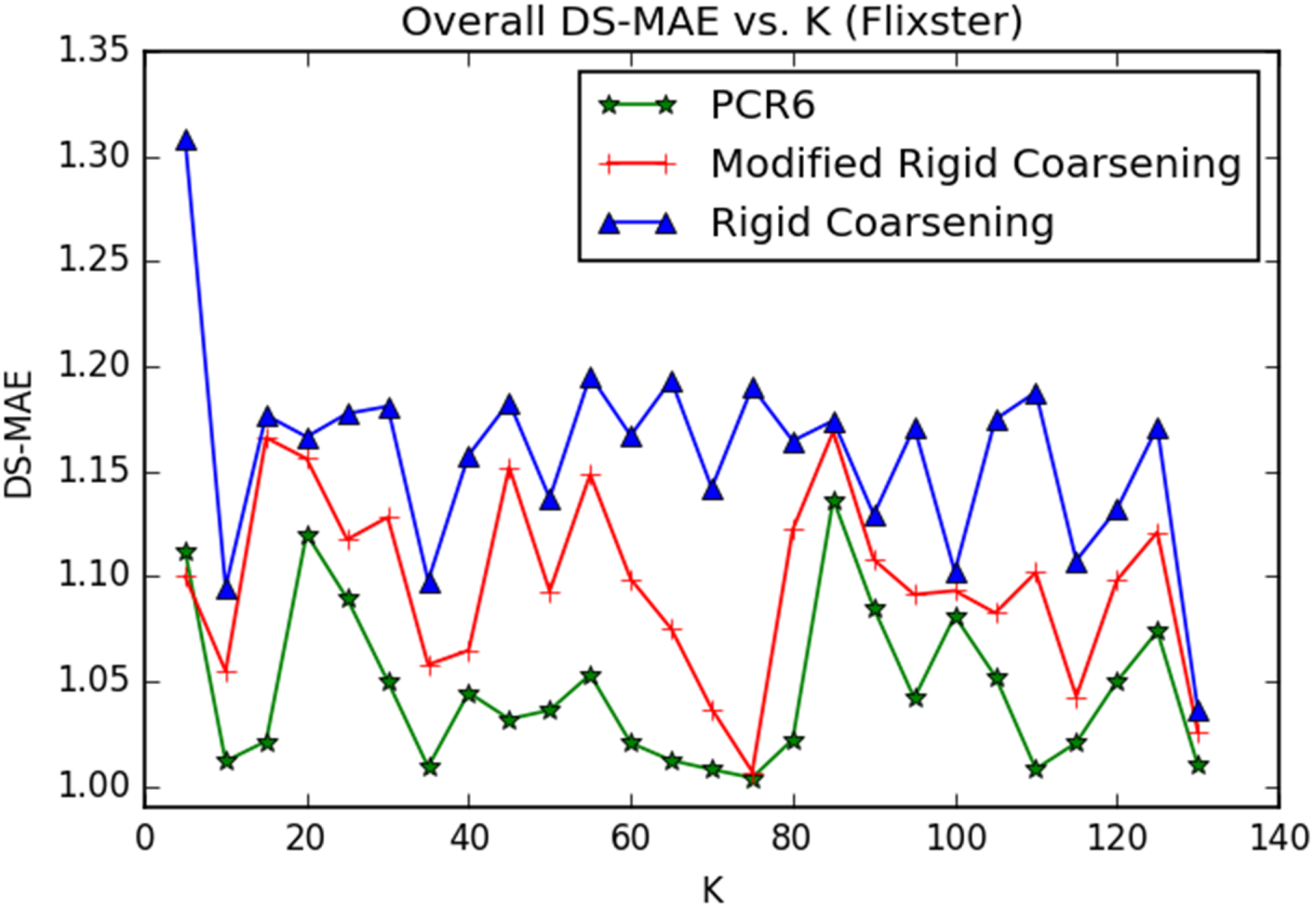
Overall DS-MAE between three combination method. (Flixster).

**Fig 13:**
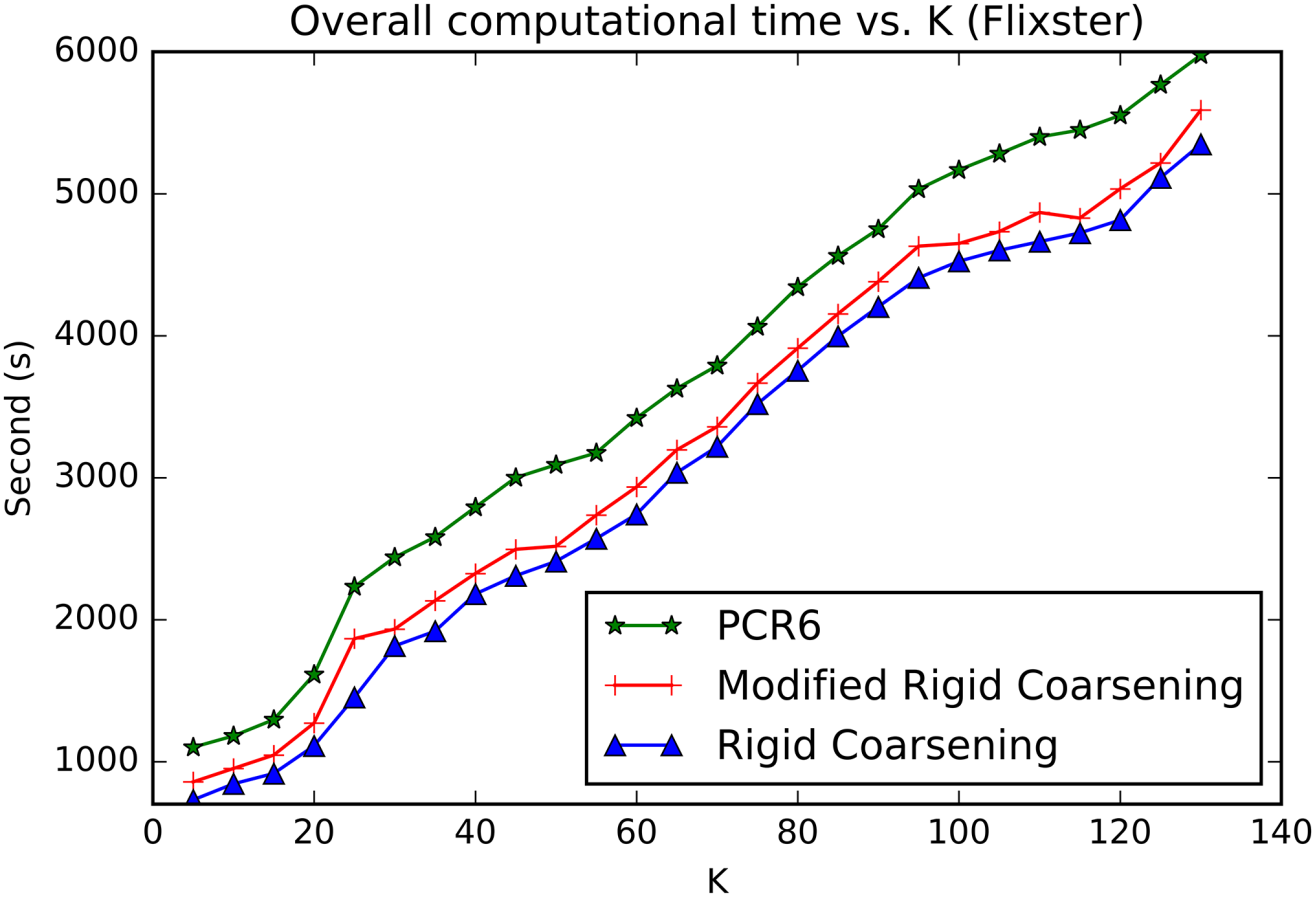
Overall computational time between three combination methods. (Flixster).

1. Experiment One:
  Movielens (http://grouplens.org/datasets/movielens) is a movie recommendation dataset widely used for benchmarking process. There are nearly 100,000 hard ratings on 19 different types of movies (Action, Comedy and so on). The domain of such rating given in Movielens includes 5 levels, denoted as Θ = {1, 2, 3, 4, 5},. At the same time, each user is required to evaluate at least 20 movies, so as to ensure adequate rating information.
  The relevant parameters used in RSs are setted: *γ* = 10^−4^ and ∀(*i*, *k*){*α*_*i*,*k*_, *σ*_*i*,*k*_} = {0.9, 2/3}. However, Setting parameter *τ* to be a fixed value is obviously unreasonable because the similarity between two users is quite different when using different combination methods. Hence, in this paper, the value of parameter *τ* will not be setted in advance. Instead, it is determined based on the similarity in matrix **S**. Specifically, the highest value of top 30% in **S** is selected for *τ*.
  Additionally, we adopt the robust strategy of 10-fold cross validation to conduct experiments, which is widely applied in experimental verification. Specific steps are as follows: original ratings in Movielens are first randomly divided into 10-folds and the experiments are thus carried out 10 times: in each sub-experiment, nine tenths of the ratings are chosen as training data and the remaining ratings are regarded as testing data. It’s worth noting that all results illustrated in the following experiments are the average values of 10 times.
  Fig 10 demonstrates the values of overall *DS-MAE* varying with changing neighborhood size *K*. And the smaller values of DS-MAE indicate the better ones. As can be seen in Fig 10, with *K* ≤ 70 performances of the three methods increase sharply as well as being the same as each other. With *K* ≥ 70, performances of both methods become stable. Especially, performance of modified rigid coarsening method is very close to classical PCR6 rules. However, original rigid coarsening is slightly worse than the other two algorithms.
  Fig 11 depicts the computational time varying with changing neighborhood size *K*. In this figure, the time taken by hierarchical coarsening combination methods (both rigid coarsening and modified rigid coarsening method) is quite faster compared to classical PCR6. Besides, modified rigid coarsening is relatively slower than original rigid coarsening. All these results illustrate that modified rigid coarsening method sacrifices some of the computational efficiency, in exchange for upgrading the accuracy of approximation.
2. Experiment Two:
  Flixster (http://www.cs.ubc.ca/jamalim/datasets/) is a classical recommendation dataset which nearly contains 535013 hard ratings on 19 different types of movies (Drama, Comedy and so on). The domain of such rating given in Flixster includes 10 levels, denoted as Θ = {0.5, 1.0, 1.5, 2.0, 2.5, 3.0, 3.5, 4.0, 4.5, 5.0},. At the same time, each user is required to evaluate at least 15 movies, so as to ensure adequate rating information. The relevant parameters used in RSs are setted: *γ* = 10^−4^ and ∀(*i*, *k*){*α*_*i*,*k*_, *σ*_*i*,*k*_} = {0.9, 2/3}. However, Setting parameter *τ* to be a fixed value is obviously unreasonable because the similarity between two users is quite different when using different combination methods. Hence, in this paper, the value of parameter *τ* will not be setted in advance. Instead, it is determined based on the similarity in matrix **S**. Specifically, the highest value of top 50% in **S** is selected for *τ*.
  Fig 12 demonstrates the values of overall *DS-MAE* varying with changing neighborhood size *K*. And the smaller values of DS-MAE indicate the better ones. As can be seen in Fig 12, we can get a similar result to the previous data set(Movielens). Especially, performance of modified rigid coarsening method is in the middle of the comparison methods. However, original rigid coarsening is worse than the other two algorithms. Fig 13 depicts the computational time varying with changing neighborhood size *K*. From this figure, we can also get the same conclusion that the time taken by hierarchical coarsening combination methods (both rigid coarsening and modified rigid coarsening method) is quite faster compared to classical PCR6.

## Conclusion

In this paper, we propose a new combination method, called modified rigid coarsening method. This new method can map the original refined FoD to the new coarsening FoD in the process of combination. Compared to traditional fusion method PCR6 in DSmT, this approach can not only reduce computational complexity, but also ensure high approximation accuracy. Besides, in order to verify the practicality of our approach, we apply this approach to fuse soft ratings in RSs. To be specific, user preferences are first transformed by DSmT-partial probability model to accurately represent uncertain information. Then, information about user preferences from different sources can be easily combined. In the future work, more helpful information will be mined to discern focal element in FoD so as to improve the accuracy of approximation and more data sets will be applied.

## Supporting information

S1 File The compressed file package of all datasets used in this paper. (RAR)

S1 Appendix Proof of *D*_1−*S*_ in Eq (9). (DOCX)
