# Supplementary material for: A fast combination method in DSmT and its application to recommender system
Source: PLoS One. 2018 Jan 19;13(1):e0189703. doi: 10.1371/journal.pone.0189703 (PMC5774721; doi:10.1371/journal.pone.0189703)
Supplement: S1 Appendix — (DOCX) [file pone.0189703.s002.docx]

S1 Appendix. Proof of $D_{1-S}$ in Equation 9

$$\begin{matrix} D_{1-S}\left(\theta_{i}\right)\triangleq\left|d_{BI}\left(m_{1}^{\Theta}\left(\cdot\right),m_{\theta_{i}}\left(\cdot\right)\right)-d_{BI}\left(m_{1}^{\Theta}\left(\cdot\right),m_{\theta_{i}}\left(\cdot\right)\right)\right| \\ \text{ +}\left|d_{BI}\left(m_{1}^{\Theta}\left(\cdot\right),m_{\theta_{i}}\left(\cdot\right)\right)-d_{BI}\left(m_{2}^{\Theta}\left(\cdot\right),m_{\theta_{i}}\left(\cdot\right)\right)\right| \\ \text{ +}\left|d_{BI}\left(m_{1}^{\Theta}\left(\cdot\right),m_{\theta_{i}}\left(\cdot\right)\right)-d_{BI}\left(m_{3}^{\Theta}\left(\cdot\right),m_{\theta_{i}}\left(\cdot\right)\right)\right| \\ \text{ }+\cdots\\ \text{ +}\left|d_{BI}\left(m_{1}^{\Theta}\left(\cdot\right),m_{\theta_{i}}\left(\cdot\right)\right)-d_{BI}\left(m_{S}^{\Theta}\left(\cdot\right),m_{\theta_{i}}\left(\cdot\right)\right)\right| \\ \text{ +}\left|d_{BI}\left(m_{2}^{\Theta}\left(\cdot\right),m_{\theta_{i}}\left(\cdot\right)\right)-d_{BI}\left(m_{1}^{\Theta}\left(\cdot\right),m_{\theta_{i}}\left(\cdot\right)\right)\right| \\ \text{ +}\left|d_{BI}\left(m_{2}^{\Theta}\left(\cdot\right),m_{\theta_{i}}\left(\cdot\right)\right)-d_{BI}\left(m_{2}^{\Theta}\left(\cdot\right),m_{\theta_{i}}\left(\cdot\right)\right)\right| \\ \text{ +}\left|d_{BI}\left(m_{2}^{\Theta}\left(\cdot\right),m_{\theta_{i}}\left(\cdot\right)\right)-d_{BI}\left(m_{3}^{\Theta}\left(\cdot\right),m_{\theta_{i}}\left(\cdot\right)\right)\right| \\ \text{ }+\cdots\\ \text{ +}\left|d_{BI}\left(m_{2}^{\Theta}\left(\cdot\right),m_{\theta_{i}}\left(\cdot\right)\right)-d_{BI}\left(m_{S}^{\Theta}\left(\cdot\right),m_{\theta_{i}}\left(\cdot\right)\right)\right| \\ \text{ }\vdots\\ \text{ +}\left|d_{BI}\left(m_{S}^{\Theta}\left(\cdot\right),m_{\theta_{i}}\left(\cdot\right)\right)-d_{BI}\left(m_{1}^{\Theta}\left(\cdot\right),m_{\theta_{i}}\left(\cdot\right)\right)\right| \\ \text{ +}\left|d_{BI}\left(m_{S}^{\Theta}\left(\cdot\right),m_{\theta_{i}}\left(\cdot\right)\right)-d_{BI}\left(m_{2}^{\Theta}\left(\cdot\right),m_{\theta_{i}}\left(\cdot\right)\right)\right| \\ \text{ +}\left|d_{BI}\left(m_{S}^{\Theta}\left(\cdot\right),m_{\theta_{i}}\left(\cdot\right)\right)-d_{BI}\left(m_{3}^{\Theta}\left(\cdot\right),m_{\theta_{i}}\left(\cdot\right)\right)\right| \\ \text{ }+\cdots\\ \text{ +}\left|d_{BI}\left(m_{S}^{\Theta}\left(\cdot\right),m_{\theta_{i}}\left(\cdot\right)\right)-d_{BI}\left(m_{S}^{\Theta}\left(\cdot\right),m_{\theta_{i}}\left(\cdot\right)\right)\right| \end{matrix}$$

$$\begin{matrix} \Rightarrow D_{1-S}\left(\theta_{i}\right)\triangleq\sum_{j=1}^{S} \left|d_{BI}\left(m_{1}^{\Theta}\left(\cdot\right),m_{\theta_{i}}\left(\cdot\right)\right)-d_{BI}\left(m_{j}^{\Theta}\left(\cdot\right),m_{\theta_{i}}\left(\cdot\right)\right)\right| \\ \text{ +}\sum_{j=1}^{S} \left|d_{BI}\left(m_{2}^{\Theta}\left(\cdot\right),m_{\theta_{i}}\left(\cdot\right)\right)-d_{BI}\left(m_{j}^{\Theta}\left(\cdot\right),m_{\theta_{i}}\left(\cdot\right)\right)\right| \\ \text{ +}\sum_{j=1}^{S} \left|d_{BI}\left(m_{3}^{\Theta}\left(\cdot\right),m_{\theta_{i}}\left(\cdot\right)\right)-d_{BI}\left(m_{j}^{\Theta}\left(\cdot\right),m_{\theta_{i}}\left(\cdot\right)\right)\right| \\ \text{ }\vdots\\ \text{ +}\sum_{j=1}^{S} \left|d_{BI}\left(m_{S}^{\Theta}\left(\cdot\right),m_{\theta_{i}}\left(\cdot\right)\right)-d_{BI}\left(m_{j}^{\Theta}\left(\cdot\right),m_{\theta_{i}}\left(\cdot\right)\right)\right| \end{matrix}$$

$$\Rightarrow D_{1-S}\left(\theta_{i}\right)\triangleq\sum_{i=1}^{S} \sum_{j=1}^{S} \left|d_{BI}\left(m_{i}^{\Theta}\left(\cdot\right),m_{\theta_{i}}\left(\cdot\right)\right)-d_{BI}\left(m_{j}^{\Theta}\left(\cdot\right),m_{\theta_{i}}\left(\cdot\right)\right)\right|$$

Due to

$$\left|d_{BI}\left(m_{i}^{\Theta}\left(\cdot\right),m_{\theta_{i}}\left(\cdot\right)\right)-d_{BI}\left(m_{j}^{\Theta}\left(\cdot\right),m_{\theta_{i}}\left(\cdot\right)\right)\right|=\left|d_{BI}\left(m_{j}^{\Theta}\left(\cdot\right),m_{\theta_{i}}\left(\cdot\right)\right)-d_{BI}\left(m_{i}^{\Theta}\left(\cdot\right),m_{\theta_{i}}\left(\cdot\right)\right)\right|$$

Thus

$$\Rightarrow D_{1-S}\left(\theta_{i}\right)\triangleq\frac{1}{2}\sum_{i=1}^{S} \sum_{j=1}^{S} \left|d_{BI}\left(m_{i}^{\Theta}\left(\cdot\right),m_{\theta_{i}}\left(\cdot\right)\right)-d_{BI}\left(m_{j}^{\Theta}\left(\cdot\right),m_{\theta_{i}}\left(\cdot\right)\right)\right|$$

End Proof.
